# A scoping review on the impact of hydrophilic versus non-hydrophilic intermittent catheters on UTI, QoL, satisfaction, preference, and other outcomes in neurogenic and non-neurogenic patients suffering from urinary retention

**DOI:** 10.1186/s12894-022-01102-8

**Published:** 2022-09-19

**Authors:** Kim Bundvig Barken, Rikke Vaabengaard

**Affiliations:** Coloplast A/S, Holtedam 1–3, 3050 Humlebaek, Denmark

**Keywords:** Intermittent catheter, Hydrophilic, Uncoated, Urinary, Bladder management, Spinal cord injury, Spina bifida

## Abstract

**Background:**

For patients suffering from urinary retention due to neurogenic [e.g., spinal cord injury (SCI), spina bifida (SB), multiple sclerosis (MS)] or non-neurogenic [e.g., cancer, benign prostate hypertrophy (BPH)] causes, intermittent catheterization is the primary choice for bladder emptying. This scoping review compared hydrophilic-coated intermittent catheters (HCICs) with non-hydrophilic (uncoated) catheters in neurogenic and non-neurogenic patients with respect to satisfaction, preference, adverse events, urinary tract infection (UTI), quality of life (QoL), cost effectiveness, pain, and discomfort.

**Methods:**

A systematic literature search was conducted using PubMed, Cochrane Library, Google Scholar, Embase, and available clinical practice guidelines and was limited to systematic reviews/meta-analysis and clinical studies (randomized trials, cohort and case–control studies) published in English between 2000 and 2020. A narrative synthesis was performed, comparing HCIC with non-hydrophilic catheters in each pathology. The articles where critically appraised and weighted according to their level of evidence based on the Oxford Centre for Evidence-Based Medicine Levels of Evidence grading.

**Results:**

Thirty seven original articles and 40 reviews were included. The comparison of HCICs versus non-hydrophilic catheters was well-documented in patients with mixed pathology, SCI, and to some extent SB. The available evidence predominantly indicates better outcomes with HCICs as reported by study authors, particularly, greater UTI reduction and improved satisfaction, cost-effectiveness, and QoL. However, SB studies in children did not report reduction in UTIs. Children complained about slippery catheters, indicating possible touching of the surface during insertion, which may compromise cleanliness of the procedure and affect outcomes such as UTI. Limited studies were available exclusively on BPH and none on MS; however, most studies performed on mixed pathologies, including BPH and MS, indicated strong preference for HCICs compared to non-hydrophilic catheters.

**Conclusions:**

The findings generally support HCICs over non-hydrophilic catheters; however, most studies were fairly small, often used a mix of pathologies, and the conclusions were often based on studies with high drop-out rates that were therefore underpowered. Larger studies are needed to support the general finding that HCICs are the preferred choice in most populations. Additional training in children or redesigned catheters may be necessary for this age-group to fully benefit from the advantages of HCICs.

**Supplementary Information:**

The online version contains supplementary material available at 10.1186/s12894-022-01102-8.

## Introduction

Urinary retention is a condition causing incomplete emptying of the urinary bladder. The prevalence of urinary retention increases with age and is typically high in neurogenic diseases. Neurogenic urinary retention can occur in case of spinal cord injury (SCI), spina bifida (SB), multiple sclerosis (MS), or other neurodegenerative diseases.

Non-neurogenic urinary retention may be caused by a physical obstruction in the urinary tract or a bladder muscle weakness [e.g., in case of cancer or benign prostate hypertrophy (BPH)] [1] or may be idiopathic.

In case of urinary retention, bladder emptying can be performed by various methods, including reflex voiding and catheterization. Several different catheterization techniques and types of catheters exist. These include indwelling catheter, suprapubic catheter, or clean intermittent catheterization as introduced by Lapides et al. [2] where a catheter is introduced through urethra and removed after bladder emptying and repeated several times a day. Intermittent catheterization is recommended for people who are able to self-catheterize [3].

Different types of urinary catheters, both hydrophilic-coated and non-hydrophilic (uncoated) catheters, are available for intermittent catheterization, with an accompanying large and heterogenous body of evidence regarding their effectiveness and applicability. Thus, a comprehensive review is needed to summarize this body of evidence, particularly one including all relevant pathologies and outcomes involved. The review presented here was performed with the aim of being wider in scope with regard to investigated outcomes than previous reviews [4–8].

## Methods

### Search strategy

The PICO used for this scoping review investigates whether, in patients with neurogenic or non-neurogenic urinary retention, better outcomes are seen with hydrophilic-coated intermittent catheters (HCICs) compared to non-hydrophilic (uncoated) catheters with respect to satisfaction, preference, adverse events, urinary tract infections (UTI), quality of life (QoL), cost effectiveness, pain, and discomfort (see Additional file 1). Such a comparison was done separately for each of the following pathologies: SC, SB, MS, BPH, bladder cancer, and mixed pathology. For the purpose of the review, HCICs were defined as catheters with a hydrophilic coating and either ready to use or requiring pre-activation with water before usage. Non-hydrophilic catheters were considered to be uncoated catheters of various materials, including catheters that are pre-lubricated.

A systematic search of the available literature was conducted in March 2022 using PubMed, Cochrane Library, Google Scholar, Embase, and available clinical practice guidelines from North America, Europe, South Africa, and Japan. The literature search strategy for the different electronic databases was based on the following search phrase developed for PubMed:

*(((((urinary) OR urethral)) AND ((intermittent catheterisation) OR (intermittent catheterization) OR (intermittent catheter))) AND (hydrophilic OR coated)*

Studies published in English between January 2000 and December 2020 were included; there were no restrictions based on age, gender, or setting. Additional articles were identified through the reference lists of the included studies.

### Eligibility criteria

The search was limited to systematic reviews/meta-analyses and clinical studies (randomized trials, controlled [non-randomized] trials, prospective and retrospective cohort studies, and case–control and repeated case–control studies). Case reports, case series, and expert opinion pieces (including commentaries, letters, and editorials) were excluded.

### Study selection

**Fig. 1:**
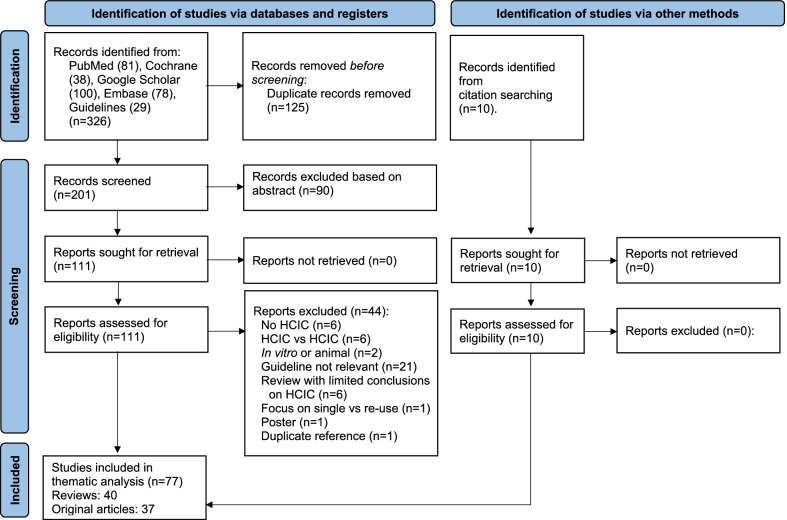
Overview of the literature search results

Once the articles were identified in each information source and duplicates removed, all abstracts in the search were read and the articles were screened for keywords (e.g., hydrophilic), and the selected outcomes before either discarding or keeping for full review. Thereafter, full reports of all titles that appeared to meet the eligibility criteria were read. At full-text stage, all clinical studies were screened for both objective data from graphs and tables describing the outcomes as well as the authors conclusions on the topics in the conclusions and discussions sections. Reviews and guidelines where only screened for conclusions on the topics. Eligible articles then progressed to data extraction, including review of references. An overview of the search procedure and results is shown in Fig. 1. Excluded studies are listed in Additional file 2 together with the reason for their exclusion.

### Data extraction and synthesis

Data were extracted and filled into a database including fixed fields and text fields, allowing filtration of the data for compiling of the scoping review. The primary data was presented in summary tables developed for this review. A narrative synthesis was performed for the included articles, comparing HCIC with non-hydrophilic catheters in each pathology (SC, SB, MS, BPH, bladder cancer, and mixed pathology) with respect to the satisfaction, preference, adverse events, UTI, QoL, pain and discomfort, cost effectiveness, as well as outcomes such as compliance/adherence, fertility, readmission, and bladder stone occurrence, by extracting the key results and conclusions concerning HCIC versus non-HCIC within the investigated outcome. PICO (population/pathology, intervention, comparison, and outcomes) tables of all the references for each outcome are listed in Additional file 1.

### Level of evidence

The included articles were weighted according to their level of evidence. This was based on the Oxford Centre for Evidence-Based Medicine (OCEBM) Levels of Evidence grading [9]. The scale ranges from 1 to 5, and only articles ranking from Level 1 to 3 were included in this summary (Levels 4 and 5, i.e., case studies, case series, and expert opinion pieces were excluded). The studies were coded with suffix A to E. Suffix A indicates a systemic review or a meta-analysis whereas B indicates an RCT, C denotes a cost effectiveness study, D an observational cohort study, a retrospective study or a self-reported questionnaire study and E covers other study designs.

### Quality of evidence

For each outcome, a table was generated with an overview of the evidence within each pathology (SC, SB, MS, BPH, bladder cancer, mixed pathology). The quality of evidence scoring was based on the available number of reviews/clinical studies at each OECBM level of evidence. A score of + + + or − − − was assigned if Level 1 studies supported the outcome, whereas + + or − − was given if multiple level 2 studies supported the outcome. If only a single Level 2 study or several Level 3 studies supported the outcome, the evidence level was ranked as + or −. The sign (±) indicates the direction of the effect according to the conclusions of the study authors; + indicates HCICs were superior to uncoated catheters, and − indicates either no significant difference between HCICs and uncoated catheters if uncoated catheters were superior).

This review presents efficacy as reported by study authors.

This review did not require ethics approval, and no protocol was enlisted in a registry.

## Results

The review presented here includes several articles [10–13] that have been overlooked by other reviews. The search identified 326 articles out of which 121 were subjected to full-text review. Of these, 77 articles [40 reviews and 37 clinical studies (i.e., original research articles)] were found to be eligible for this literature summary (Fig. 1). An overview of the evidence level for the different outcomes and pathologies are shown in Table 1. In general, all the studies included compare hydrophilic catheters (both specified and unspecified) to non-hydrophilic, including both prelubricated and unlubricated catheters that require manual lubrication. Catheterization was through urethra, but a few studies also included some patients with abdominal wall catheterizable channels. We included studies with participants of all ages, pathologies (neurogenic and non-neurogenic) and gender. The articles included cover a wide range from meta and systemic reviews and various types of clinical studies, both RCT’s, but also other studies such as cost effectiveness studies, observational cohort studies, retrospective studies and studies based on self-reported questionnaires. Many studies were of low quality and many of the RCT’s had few participants and high drop-out rates and often they did not meet the calculated sample size. In the 18 RCTs that were included, the study period varied from 1 to 24 months and the number of participants from 25 to more than 200.

**Table 1:** Overview of the evidence level for a comparison of HCIC and non-HCIC catheters for the different pathologies

Population	Satisfaction	Preference	Adverse events	UTI	QoL	HEOR	Pain and discomfort
SCI	+ +/−	+	+ +/− −	+ + +/−	+ +	+ + +/− −	+
SB	+/− −	+ +/−	/− −	+/− −	+ +	NA	/−
MS	NA	NA	NA	NA	NA	NA	NA
BPH	NA	NA	NA	NA	NA	NA	NA
Mixed	+ + +/−	+ + +	+ + +/−	+ + +/− −	+ + +	+ + +/− −	+ + +/−
All	+ + +/−	+ + +	+ + +/− −	+ + +/− −	+ + +	+ + +/− −	+ + +/− −

*BPH* Benign prostate hypertrophy, *HCIC* Hydrophilic-coated intermittent catheters, *HEOR* Health economics and outcomes research, *MS* Multiple sclerosis, *NA* Not available, *QoL* Quality of life, *SB* Spina bifida, *SCI* Spinal cord injury, *UTI* Urinary tract infection

+ to + + +: The literature supports claims of hydrophilic catheters as being superior to uncoated catheters

− to − − − No significant difference between hydrophilic and uncoated catheters or uncoated catheters are superior

### Satisfaction

Satisfaction was typically evaluated by questionnaires, some studies using interview-type questions and others using scores based on scales such as the visual analogue scale (VAS). Table 2 gives an overview of the available literature and evidence level pertaining to satisfaction. Overall, 28 articles (19 original articles and 9 reviews) were found covering satisfaction either as the primary or the secondary research parameter. Of these, 21 studies favored HCICs over non-hydrophilic catheters for improvement in satisfaction.

**Table 2:** Satisfaction evidence level

Population	Evidence Pro/con HCIC	Original studies (n)	Reviews (n)	LoE
SCI	+ +/−	5	1	2A-3B
SB	+/− −	5	NA	2B
MS	NA	NA	NA	NA
BPH	+/−	NA	NA	NA
Mixed	+ + +/−	9	8	1A-2B
**All**	**+ + +/−**	**19**	**9**	**1A-2B**

Bold text is a summary of the total evidence level of the pathologies

*BPH* Benign prostate hypertrophy, *HCIC* Hydrophilic-coated intermittent catheters, *LoE* Level of evidence, *MS* Multiple sclerosis, *NA* Not available, *SB* Spina bifida, *SCI* Spinal cord injury

+ to + + +: The literature supports claims of hydrophilic catheters as being superior to uncoated catheters

− to − − No significant difference between hydrophilic and uncoated catheters or uncoated catheters are superior

1, 2, and 3 refer to the (descending) level of evidence. A = systematic review/meta-analysis; B = clinical trial

#### Spinal cord injury

The corresponding literature (6 studies, Table 2) indicates that, in SCI patients, satisfaction is generally higher with HCICs (such as SpeediCath^®^, Rüsch^®^, or LoFric^®^) than non-HCICs (lubricated polyvinyl chloride [PVC] catheters or undefined). For example, Cardenas [14] found a significantly higher satisfaction rate with HCIC (SpeediCath) than gel-lubricated PVC catheters (Conveen^®^). Wyndaele [15] evaluated patient satisfaction using VAS scores, and like the Cardenas study, observed increased satisfaction with HCICs. Spinu [12] also observed significantly higher satisfaction with HCICs than non-HCICs. Johansen et al. [16] reported increased general satisfaction with HCICs and that 74% of the patients wanted to continue with their hydrophilic catheters (LoFric). In contrast, Sarica [17], who measured satisfaction using VAS scores, found that gel lubricated catheters scored significantly higher than HCICs, which in turn, scored higher than uncoated catheters in satisfaction. In a review, Salameh et al. [18] concluded that the use of HCICs is an innovative technique that leads to better patient comfort and satisfaction in SCI patients.

#### Spina bifida

Five studies concerned SB patients, two of which showed higher satisfaction with HCICs (DeFoor [19] and Burki [11]) whereas the others either showed higher satisfaction for PVC (Kiddoo [20], Chick [21]) or revealed difficulties inserting the catheter despite being coated or not (Lindehall [13]).

#### Multiple sclerosis

No information on satisfaction in MS patients was found.

#### Benign prostate hypertrophy

Only one article investigated satisfaction among BPH patients, which did not find any difference in satisfaction between HCICs and a lubricated (non-hydrophilic) Mentor catheter (Pachler and Frimodt [22]). However, this article was not part of the literature search analysis as it was published before 2000.

#### Bladder cancer

Cindolo [23] found that among bladder cancer patients, HCIC users had significantly more comfort than users of non-coated catheters; the analysis was based on VAS scores. Compared to some of the neurogenic disorders, this patient group may, to a higher degree, have full sensation, affecting results on satisfaction.

#### Mixed population

Nine studies and eight reviews were performed on mixed pathology patient groups. Most studies were in favor of HCICs with regard to satisfaction [1, 6, 10, 24–32]. A few studies had ambiguous results [4, 33, 34], and one study did not find any difference; in this study, Yamanishi et al. [35] compared SpeediCath Compact (HCIC) with standard non-coated nelaton catheters in males and found no statistically significant difference in terms of ease of use, convenience, discreetness, and psychological well‐being between the two catheter types. However, there were significant differences in favor of SpeediCath Compact in terms of storage of catheters at home and the response that the “catheter allows me to feel confident when away from home,” although these scores are probably more related to the fact that the HCIC was compact. Stensballe et al. [25] performed a study on healthy males to test withdrawal force, pain, and preference for different types of catheters. SpeediCath exerted significantly lower withdrawal friction force compared to LoFric (also hydrophilic) and gel lubricated PVC catheters; moreover, SpeediCath and LoFric caused less hematuria and pain than the non-hydrophilic PVC catheter. Newman et al. [32, 36] presented an increase in mean Intermittent Self-Catheterization Questionnaire (ISC-Q) score from 58.00 to 67.19 when patients switched to single-use HCICs. This corresponds to a statistically significant change of 9.42 units (*p* = 0.0101) and a 20% increase in health-related QoL. In a population of female neurogenic patients, comparing hydrophilic catheters (SpeediCath) with uncoated catheters, Yoshida et al. [10] found that the hydrophilic catheter was considered significantly more safe than the uncoated catheter, and satisfaction with the hydrophilic catheter was significantly higher (13/16 vs 3/13, *p* = 0.003).

### Preference

In total, 15 articles were found discussing preference (Table 3). Only one article, Kiddoo et al. [20], found that participants did not favor the use of HCICs. The results of all the other articles were, to varying degrees, positive towards HCICs in terms of patient preference.

**Table 3:** Preference evidence level

Population	Evidence Pro/con HCIC	Original studies (n)	Reviews (n)	LoE
SCI	+	2	NA	2B
SB	+ +/−	4	NA	2B-3B
MS	NA	NA	NA	NA
BPH	NA	NA	NA	NA
Mixed	+ + +	7	2	2A-2B
**All**	**+ + +**	**13**	**2**	**2A-2B**

Bold text is a summary of the total evidence level of the pathologies

*BPH* Benign prostate hypertrophy, *HCIC* Hydrophilic-coated intermittent catheters, *LoE* Level of evidence, *MS* Multiple sclerosis, *NA* Not available, *SB* Spina bifida, *SCI* Spinal cord injury

+ to + + +: The literature supports claims of hydrophilic catheters as being superior to uncoated catheters

− No significant difference between hydrophilic and uncoated catheters or uncoated catheters are superior

1, 2, and 3 refer to the (descending) level of evidence. A = systematic review/meta-analysis; B = clinical trial

#### Spinal cord injury

Both articles in this disease category, Pinder [37] and Johansen [16], found a higher preference for HCICs than lubricated PVC catheters in SCI patients.

#### Spina bifida

In SB patients, DeFoor [19] and Burki [11] both noticed a significantly higher preference for HCICs after the respective trials, whereas Chick [21] noted that adaption to HCICs was difficult; however, those who got it to work, preferred HCICs. Kiddoo [20] found that fewer participants in the HCIC group (57%) answered “Yes” to “Would you continue using the product?” compared to the PVC catheter group (92%); however, the authors also stated that the issue was related to the initial difficulty in handling the hydrophilic catheter by the children due to the slipperiness. Lucas [38] recorded increased preference for HCICs versus uncoated PVC catheters in myelomeningocele patients.

#### Multiple sclerosis

No studies were found concerning preference for HCICs in MS patients.

#### Benign prostate hypertrophy

A study by Pachler and Frimodt in 1999 on BPH comparing HCICs (LoFric) with uncoated PVC catheters, which was not part of the analysis, found a slightly higher preference for HCICs (15/32) compared to the uncoated catheters (11/32); however, the difference was not statistically significant.

#### Mixed pathology

In mixed pathology patients, Vapnek et al. [27] performed a crossover study comparing HCICs (LoFric; single-use) with uncoated PVC catheters (reuse) and found that patients preferred the HCICs because of ease of use. This was also observed by Sutherland [39] (not included in the analysis but found to be relevant) and Stensballe [25] who both studied male subjects, and found that, respectively, 81% and 93% of the subjects preferred HCICs over uncoated catheters. Johansen [16] reported that patients prefer to use HCICs versus non-HCICs and that HCICs should be regarded as the gold standard for intermittent catheterization. Newman et al. [32, 36] found that at the end of their study in which patients using reusable non-hydrophilic catheters switched to single-use HCICs, 83% preferred to continue with the single-use HCICs. Yoshida et al. [10] compared SpeediCath hydrophilic catheters to uncoated catheters in women with neurogenic diseases. The willingness to use a hydrophilic catheter was significantly higher compared to an uncoated catheter (15/16 vs 6/13, *p* = 0.01). Similar conclusions were found by Leriche et al. [30] and Hedlund et al. [28] and in the review by Shamout et al. [6], the conclusion was the same. Interestingly Boucher et al. [34] came to a different conclusion, finding that after a short trial period, most children preferred their uncoated catheter and would not change to the HCIC. Only 10 patients (33%) would be ready to change their actual catheter for the HC. Of these patients, 1 was a male (by urethra) and 9 were females.

### Adverse events

A total of 31 articles, most of them reviews (n = 19), have discussed adverse events (Table 4). The term adverse events cover a wide spectrum from hematuria, bleeding, urethral irritation, inflammation, and damage. Conflicting conclusions exist with at least 21 articles showing reduction in adverse events with HCICs while 8 either show no difference or reduced number of adverse events in the control (non-HCIC) group.

**Table 4:** Adverse events evidence level

Population	Evidence Pro/con HCIC	Original studies (n)	Reviews (n)	LoE
SCI	+ +/− −	5	5	2A-2B
SB	/−	4	NA	2B
MS	NA	NA	NA	NA
BPH	NA	NA	NA	NA
Mixed	+ + +/−	3	14	1A-2B
**All**	**+ + +/− −**	**12**	**19**	**1A-3B**

Bold text is a summary of the total evidence level of the pathologies

*BPH* Benign prostate hypertrophy, *HCIC* Hydrophilic-coated intermittent catheters, *LoE* Level of evidence, *MS* Multiple sclerosis, *NA* Not available, *SB* Spina bifida, *SCI* Spinal cord injury

+ + to + + +: The literature supports claims of hydrophilic catheters as being superior to uncoated catheters

− to − − No significant difference between hydrophilic and uncoated catheters or uncoated catheters are superior

1, 2, and 3 refer to the (descending) level of evidence. A = systematic review/meta-analysis; B = clinical trial

#### Spinal cord injury

Five clinical studies and five reviews discussed adverse events in SCI patients. In general, the results point in different directions. Spinu [12] found a significantly lower number of post/intra/inter-catheterization bleeding episodes, and Cardenas [14] found a significant reduction in hematuria in the HCIC group (23%) versus the group using uncoated catheters (34%); however, the number of urethral bleeding episodes in the HCIC group was significantly higher than that in the uncoated catheters group (*p* < 0.05). On the contrary, De Ridder [24] did not observe any difference in hematuria, and Sarica [17] found that gel lubricated catheters reduced urethral microtrauma and pyuria while at the same time reducing hematuria when compared with HCICs. Samal [40] found no difference in urethral trauma between HCICs (SpeediCath) and standard PVC catheters; however, the lesions resulting after using PVC catheters were of greater extent. Most of the studies were affected by high drop-out rates making conclusions difficult. The five available reviews [8, 18, 41–43] all favored HCICs over standard catheters with respect to reducing adverse events.

An older (pre-2000) retrospective long-term study performed by Waller et al. [44] investigated urethral complications. Urethral damage was found in the study; however, it was induced before catheterization with HCIC (LoFric) was begun, and no urethral trauma was identified during the 5–9 year follow-up period.

#### Spina bifida

Four clinical studies on adverse events in SB patients were found (DeFoor [19], Burki [11], Kiddoo [20] and Lindehall [13]). None of the studies observed any significant difference between HCICs and non-HCICs with regard to hematuria. All four studies were performed on children, which may affect the outcome when compared with SCI patients, for whom all the studies were on adults.

#### Multiple sclerosis and benign prostate hypertrophy

No studies on adverse events focusing exclusively on MS or BPH patients were found.

#### Mixed pathology

Fourteen reviews and guidelines and three studies were on mixed pathologies. Of the three studies, both Stensballe [25] and Vapnek [27] measured significantly less microhematuria with the hydrophilic catheter and Leriche [30] experienced 5 episodes of bleeding with a gel coated catheter compared to no episodes with the hydrophilic catheter. Among the 14 reviews and guidelines, 11 concluded that hydrophilic catheters reduced the risk of microhematuria, urethral irritation, inflammation, and damage [1, 4, 6, 28, 29, 31, 45–49]. Two reported that HCIC may be preferable to standard non-coated catheters, but that uncoated catheters may be just as good [26, 50]; Rognoni et al. [5] reported that a risk reduction for hematuria associated with hydrophilic-coated catheters in general was not demonstrated.

### Urinary tract infection

UTI is the most researched field concerning the efficacy of HCICs versus uncoated catheters; yet, it remains the area with the most contradictory findings. In general, the research community is divided into two groups: those that demonstrate reduced UTIs related to the use of HCICs and those who question the benefits of HCICs related to reduced UTI. Another complicating factor is the various UTI definitions and UTI frequency metrics used in the literature.

#### UTI definition

There is no universally accepted definition of UTI in individuals with neurogenic lower urinary tract dysfunction. Most studies use either (i) a symptomatic definition of UTI (i.e., antibiotic treatment has been prescribed) or (ii) a strict definition of symptomatic UTI [14]:

- Antibiotic treatment has been prescribed.
- Bacteriuria ≥ 10^2^ CFU/mL.
- At least one of seven UTI symptoms based on consensus guidelines (fever, autonomic dysreflexia [sweating, bradycardia, blood pressure elevation], increased spasticity, discomfort or pain over the kidney or bladder or during micturition, onset and/or increase in incontinence episodes, cloudy urine with increased odor, malaise, lethargy, or sense of unease).
- Dipstick test is positive for leukocyte esterase.

However, other studies use either self-reported UTI, bacteriuria, or other definitions, making direct comparisons or meta studies complicated. For this study, we have not defined any exclusion or inclusion criteria regarding UTI definition; however, it could be argued that for catheter-associated UTIs, a higher level of bacteriuria should be accepted like the catheter-associated UTI definition from the 2009 International Clinical Practice Guidelines from the Infectious Diseases Society of America [51]. In general, it must be assumed that UTIs within both neurogenic and non-neurogenic patients suffering from urinary retention are either catheter associated or a result of incomplete bladder emptying.

#### UTI frequency

The frequency of UTI has been reported differently in the different studies, either as the percent of affected patients within the study period, total number of events in the study period, number of patients with two or more UTIs (i.e., recurrent UTI), or the average number of events/year.

In this review, a total of 20 clinical studies and 32 reviews and guidelines were found that investigated if hydrophilic catheters reduce UTI frequency when compared with uncoated catheters (Table 5). Ten out of the 20 clinical studies included numbers that could be extracted and compared (Table 6), although some presumptions like UTI frequency measures needed to be made. Among these 10 studies, all but two studies found a reduction in UTIs when using HCIC (however, not all findings where significant). The two studies that concluded differently used participants below 18 years of age. In all of the studies [3] that had patients below 18 years, the patients complained that the hydrophilic catheter was too slippery, which indicates that the catheters are touched on the sterile surface before insertion, which may explain the contradictory outcome related to UTI seen in two of these studies.

**Table 5:** Urinary tract infection

Population	Evidence Pro/con HCIC	Original studies (n)	Reviews (n)	LoE
SCI	+ + +/−	12	10	1A-2B
SB	+/− −	4	NA	2B-3B
MS	NA	NA	NA	NA
BPH	–	NA	NA	NA
Mixed	+ + +/− −	4	22	1A-2B
**All**	**+ + +/− −**	**20**	**32**	**1A-2B**

Bold text is a summary of the total evidence level of the pathologies

*BPH* Benign prostate hypertrophy, *HCIC* Hydrophilic-coated intermittent catheters, *LoE* Level of evidence, *MS* Multiple sclerosis, *NA* Not available, *SB* Spina bifida, *SCI* Spinal cord injury

+ to + + +: The literature supports claims of hydrophilic catheters as being superior to uncoated catheters

− to − − No significant difference between hydrophilic and uncoated catheters or uncoated catheters are superior

1, 2, and 3 refer to the (descending) level of evidence. A = systematic review/meta-analysis; B = clinical trial

**Table 6:** Clinical studies with available data for a comparison of urinary tract infection (UTI)

Author, year	No. of patients	HCIC	UC	UTI definition	Period (months)	UTI frequency	Significant difference	Risk ratio
Cardenas 2009 [52]	45	12/22	14/23	Symptomatic	12	1 or more	No	0.9
Cardenas 2011 [14]	188	69/91^a^	85/97^a^	Symptomatic	6	1 or more	No^b^	0.87
De Ridder 2005 [24]	123	39/61	51/62	Symptomatic	12	1 or more	Yes	0.78
Sutherland 1996 [39]	30	1/16	1/14	Symptomatic	NA	1 or more	No	0.88
DeFoor 2017 [19]	55	2/22	7/33	Strict^c^	12	1 or more	Yes	0.43
Samal 2011 [40]	53	28/36	15/17	Symptomatic	24	1 or more	Yes^d^	0.88
Yoshida 2018 [10]	29	2/16	5/13	Symptomatic	6	1 or more	No	0,33
Cindolo 2004 [23]	100	3/50	7/50	Symptomatic	24	2 or more	Yes	0.43
Burki 2019 [11]	101	17/48	12/53	Symptomatic	12	2 or more	No	1.56
Samal 2011 [40]	53	5/36	7/17	Symptomatic	24	2 or more	Yes^d^	0.33
Kiddoo 2015^e^ [20]	45	3,42	2,2	Symptomatic	5.5	Person weeks of UTI	Yes^f^	1.55

*HCIC* Hydrophilic-coated intermittent catheterization, *UC* Uncoated catheter, *UTI* Urinary tract infection

^a^Estimated from curves by Rognoni [5]

^b^Not significant, but the time to first UTI was found to be significant

^c^Strict definition of UTI includes:

i. Antibiotic treatment has been prescribed

ii. Bacteriuria ≥ 10^2^ CFU/mL

iii. At least 1 of 7 UTI symptoms based on consensus guidelines [fever, autonomic dysreflexia (sweating, bradycardia, blood pressure elevation), increased spasticity, discomfort or pain over the kidney or bladder or during micturition, onset and/or increase in incontinence episodes, cloudy urine with increased odor, malaise, lethargy, or sense of unease]

iv. Dipstick test is positive for leukocyte esterase

^d^Average number of infections is significant

^e^HCICs versus non-HCIC (both single use and reuse), measuring weeks with UTI

^f^Claim a *p*-value of < 0.001; however, based on the overlapping confidence intervals in the original data, the significance of the difference is questionable

#### Spinal cord injury

For SCI patients, Cardenas et al. [14] found a 33% decrease in the daily risk of UTI among patients using HCICs, and the time to the first antibiotic-treated symptomatic UTI was significantly delayed in the HCIC group [14]. In another study by Cardenas et al. from 2009, increased incidence of UTI in the control group than in the HCIC group was also noted, although this difference was not statistically significant [52]. De Ridder et al. [24] also found that significantly fewer individuals using HCICs experienced UTI compared to non-HCIC users. However, this was only in a male population and the study results may have been affected by a high drop-out rate. Samal et al. [40] conducted a long-term study and found significantly lower incidence of symptomatic UTIs in the group using hydrophilic catheters (SpeediCath): 0.94 versus 1.58 (*p* < 0.05). Afsar [53] also saw approximately one UTI per year in the group using HCIC versus two UTIs annually in the control group (*p* = 0.05). Contradicting these results, Wyndaele [15] did not find any difference in UTI incidence between HCIC and non-HCIC users. However, the experiment was only followed up for one month. A short follow up period is also seen in the other two studies showing little difference between HCICs and non-HCICs (Massa [54], Samal [55]), in which patients were only followed for up to three months. Several other studies also reported reduction of UTIs in the hydrophilic group, although not statistically significant [12, 17, 56, 57].

Ten reviews discussed the effect of switching to HCICs and its influence on UTI frequency in SCI patients. Two reviews (Bermingham [58] and Nicolle [59]) concluded that the influence of HCICs on the frequency of UTIs remains controversial, and based on the available studies, these reviews concluded that HCICs did not affect the frequency of UTI. However, three other newer reviews concluded in favor of HCICs for the reduction of UTI frequency (Christison [60], Salameh [18] and Li [8]). Li et al. calculated odds ratios (ORs) rather than RRs and made erroneous calculations concerning the number of events and participants in the Cardenas [14] study, resulting in a too positive review in favor of HCIC. The newest guidelines for best practices for bladder management in SCI in South Africa [42] also claim that HCICs reduce the risk of UTI. Overall, the amount and level of evidence supports HCICs over non-HCICs in SCI patients in terms of reduction of UTI incidence. D’Hondt and Everaert 2011 claimed that HCIC reduced UTIs [41], whereas the reviews by Hill [61] and Romo [62] reported that data so far is controversial and that more data is needed. In the guidelines from SIU-ICUD on urologic management of neurogenic bladder after spinal cord injury, they state that HCIC may be offered to SCI patients with recurrent complications [63]. The HICPAC guideline for catheter associated UTIs note that hydrophilic catheters might be preferable to standard catheters for patients requiring IC [64]. In a review of 5 guidelines Tambyah discuss the general lack of mentioning of HCIC and that only HICPAC promote HCIC [65].

#### Spina bifida

Four clinical studies have been conducted in patients with SB. DeFoor [19] compared HCICs (LoFric) with lubricated PVC catheters for a period of 12 months and found a significant reduction in UTI rate in the HCIC group (2/22 UTIs/person-year) versus the uncoated group (17/33); however, both Burki [11] and Kiddoo [20] found that HCICs actually increased the risk of UTI. Nevertheless, all three studies suffered from high drop-out rates, and at least one study, Kiddoo 2015, was underpowered; moreover, in the Burki study from 2019, the two groups did not exclusively stick to either HCIC or PVC, but instead used a combination. The groupings were done retrospectively to the predominant catheter type. The three studies were all conducted on children. A fourth study by Lucas et al. [38] investigated both children and adults with myelomengiocele and found the same frequency of UTI, but higher number of samples positive of pathogenic bacteria in the uncoated versus the HCIC group; however, the results were not significant.

#### Multiple sclerosis

No studies were conducted in MS patients.

#### Non-neurogenic population (benign prostate hypertrophy and bladder cancer)

Only two studies in non-neurogenic patients were found. Cindolo et al. [23] investigated UTI frequency in cancer patients using intermittent catheterization. They found that HCICs were associated with a significantly lower occurrence of UTI (3.5% vs 7.4%; *p* < 0.01). The other study (not included in the analysis) investigated UTIs in patients with BPH (Pachler and Frimodt-Møller [22]) but did not find any advantages of using HCICs (LoFric) versus uncoated PVC catheters with regard to bacteriuria; however, it was a small crossover study, with patients being on each catheter for only three weeks.

#### Mixed pathology

In total, 22 reviews and guidelines on UTI frequency related to HCICs were found on patients with mixed pathology. Most of the newer reviews are a response to the 2014/17 Cochrane review [26, 66]. In the Cochrane meta review, results favored HCICs, although not being statistically significant. Authors concluded that uncoated catheters are similar to HCIC in terms of UTI frequency. A number of reviews with high levels of evidence have reached a different conclusion, primarily based on the clinical studies by Cardenas [14, 52] and De Ridder [24]. Feng et al. [4] found an OR of 54% in favor of HCICs versus non-HCICs. Rognoni [5] also showed a significantly reduced RR of developing UTI when using HCICs. Shamout [6] concluded that although there are inconsistent results in the literature, the use of single-use hydrophilic catheters minimizes the risk of UTI. Kennelly [48], Biardeau [67], and Selius [1] all concluded that less UTIs are associated with hydrophilic catheters when compared with uncoated catheters. The European Association of Urology Nurses (EAUN) guidelines recommend that single-use aseptic technique should be used to reduce UTIs, and state that there is a high level of evidence that hydrophilic coating reduces the risk of UTI [47]. The European Association of Urology (EAU) guidelines recommend aseptic technique and sterile lubricated or hydrophilic catheters to be used to prevent UTI [68]. The Ontario Medical Advisory Secretariat from 2006 contradict these conclusions and state that there is insufficient evidence to indicate that hydrophilic catheters are associated with a lower rate of UTIs [33], which is along the lines of the Cochrane reviews of 2007 [69], 2014 [26] and 2017 [66] (retracted). The recent Australian guideline for intermittent catheterization (2019) [70] also concluded that there is currently no established comparative benefit between the catheter types in terms of reducing the risk of UTIs. From the 6th International Consultation on Incontinence session in Tokyo [29], the editorial board concluded that the evidence to suggest one specific catheter type is weak and that the choice of catheters and regimens should be made on an individual basis. Several other reviews on mixed pathology also concluded that HCIC was associated with reduced risk of UTI and bacteriuria [28, 45, 48, 71, 72], and Madersbacher in 2017 stated that coated catheters have yielded better results in a series of studies compared to uncoated catheters, although clear evidence is not present due to the design of the studies undertaken [73].

In the clinical study by Yoshida et al. [10], 36 female patients with neurogenic bladder were randomized into two groups, testing a hydrophilic-coated compact catheter (SpeediCath Compact) versus an uncoated standard catheter. Over a 6 month period, the number of symptomatic UTIs was 2/16 UTIs in the HCIC group and 5/13 in the non-hydrophilic catheter group; the difference was not statistically significant. Vapnek in 2003 found a significantly larger drop in UTI frequency in HCIC versus PVC (re-use), but from a higher starting point and the end frequency was the same for both catheters [27]. This study was performed on 36 patients. In a questionnaire-based retrospective study with 694 respondents, Neovius in 2015 found that switching to HCIC reduces infections/UTI [74].

### Quality of life

QoL has only been evaluated in a few studies (n = 8, Table 7), and often it was done with non-validated questionnaires; the questionnaires also used dissimilar scoring systems like VAS scores and numerical scores.

**Table 7:** Quality of life

Population	Evidence Pro/con HCIC	Original studies (n)	Reviews (n)	LoE
SCI	+ +	NA	2	2A-3A
SB	+ +	1	NA	2B
MS	NA	NA	NA	NA
BPH	NA	NA	NA	NA
Mixed	+ + +	3	2	2A-2B
**All**	**+ + +**	**4**	**4**	**2A-2B**

Bold text is a summary of the total evidence level of the pathologies

*BPH* Benign prostate hypertrophy, *HCIC* Hydrophilic-coated intermittent catheters, *LoE* Level of evidence, *MS* Multiple sclerosis, *NA* Not available, *SB* Spina bifida, *SCI* Spinal cord injury

+ + to + + +: The literature supports claims of hydrophilic catheters as being superior to uncoated catheters

2 and 3 refer to the (descending) level of evidence. A = systematic review/meta-analysis; B = clinical trial

#### Spinal cord injury

Only one review and one practice guideline discussed QoL in relation to the use of HCICs in SCI patients. The articles are based primarily on clinical trials that were not exclusively performed on SCI patients but also in patients with neurogenic bladder in general. D’Hondt [41] concluded that HCICs may be preferable to uncoated PVC catheters in terms of QoL. The South African guideline [42] states that single-use hydrophilic-coated catheters increase social participation by saving time, increasing independence, and reducing the burden related to clean intermittent catheterization, thereby improving QoL.

#### Spina bifida

Only one clinical trial was found on SB patients with regard to QoL; DeFoor [19] concluded that HCIC patients reported a decrease in discomfort with the catheterization process, from baseline (1.7) to the end of the study (0.4), on a rating scale ranging from 0 (no discomfort) to 10 (maximal discomfort); however, the drop in discomfort was not statistically significant (*p* = 0.06) and the questionnaire used was not previously validated; thus, the clinical relevance of the identified difference is unknown.

#### Multiple sclerosis and benign prostate hypertrophy

No studies on MS or BPH patients were found.

#### Mixed pathology

Newman et al. [36] found that the mean ISC-Q score increased from 58.00 to 67.19 when patients switched to the single-use HCIC (*p* = 0.0101), corresponding to a 20% increase in health-related QoL. In the clinical study by Yoshida et al. [10], after a 6 month period, the participants answered both an ISC-Q and a Qualiveen^®^ questionnaire; the hydrophilic group scored significantly higher on 3 out of 4 subscales of the ISC-Q, including ease of use, convenience, and well-being (*p* < 0.05), and on all 4 subscales of the Qualiveen questionnaire (*p* ≤ 0.006). Chartier-Kastler and Denys in 2011 concluded that the available data indicates that hydrophilic catheters may be preferable to PVC catheters in terms of safety and QoL [31]; in a review by Shamout et al. [6], evidence supported the benefits of hydrophilic-coated catheters in terms of QoL, and 7 out of the 9 studies investigated reported significant improvement in satisfaction and preference with HCICs versus uncoated catheters, with the two last studies reporting an insignificant difference in these parameters between the two catheter types.

### Health economics and outcomes research

Several studies have discussed results from a health economics and outcomes research (HEOR) perspective. The calculations were based on the proposed preventative effects on UTI from using hydrophilic-coated catheters versus uncoated catheters and the related cost savings from fewer lifetime hospital days and medication versus the higher price of a hydrophilic-coated catheter. The calculations are often country specific because the value of quality-adjusted life years (QALYs) vary from country to country.

In total, 12 studies were found on HEOR comparing HCICs with non-HCICs (Table 8). The studies are not directly comparable as they are based on the individual country’s economy and reimbursement plans.

**Table 8:** Health economics and outcomes research

Population	Evidence Pro/con HCIC	Original studies (n)	Reviews (n)	LoE
SCI	+ + +/− −	6	1	1A-3B
SB	NA	NA	NA	NA
MS	NA	NA	NA	NA
BPH	NA	NA	NA	NA
Mixed	+ + +/− −	2	3	1A-2B
**All**	**+ + +/− −**	**8**	**4**	**1A-3A**

Bold text is a summary of the total evidence level of the pathologies

*BPH* Benign prostate hypertrophy, *HCIC* Hydrophilic-coated intermittent catheters, *LoE* Level of evidence, *MS* Multiple sclerosis, *NA* Not available, *SB* Spina bifida, *SCI* Spinal cord injury

+ + +: The literature supports claims of hydrophilic catheters as being superior to uncoated catheters

− No significant difference between hydrophilic and uncoated catheters or uncoated catheters are superior

1, 2, and 3 refer to the (descending) level of evidence. A = systematic review/meta-analysis; B = clinical trial

#### Spinal cord injury

Seven articles concerning HEOR in SCI patients were found (Table 8). The only review available for SCI patients (Bermingham [58]) is a study to support the UK National Institute for Health and Care Excellence (NICE) guidelines [75]. The calculated incremental cost-effectiveness ratio (ICER) was £ 54,350.00 for pre-lubricated versus uncoated (re-use) catheters, which was above the limit of being cost effective. HCICs were found to be more expensive and less effective when compared with pre-lubricated catheters (based only on the non-significant UTI reduction observed by Cardenas [52]). Based on the concerns of re-use of single use medical devices, the NICE guideline ended up recommending that people should be given a choice between single-use hydrophilic-coated or gel reservoir catheters, despite the findings in the study.

Six other studies were conducted on SCI patients, all concluding in favor of HCIC being the more cost-effective solution. Clark et al. [76] concluded the opposite of Bermingham [58] for the UK, finding that the use of HCICs in SCI patients is highly cost effective: + 1.4 life years gained and a 16% reduction in UTIs, with an ICER of £ 6100.00 per QALY gained, which is within the NICE cost-effectiveness threshold. For Italy, Rognoni et al. [77] concluded that, when considered over a lifetime, HCICs are a potentially cost-effective choice in comparison with uncoated catheters, as the ICER was below the Italian threshold for being cost-effective. For Brazil, Truzzi [78] found that despite a difference in unit cost of the two different types of catheters, the hydrophilic-coated catheters seem to be cost-effective for SCI patients from a lifetime perspective. Welk et al. [79] suggested that reimbursement of HCICs should be considered in SCI patients in Canada. They found an ICER of CAD $ 66,634/QALY, which is below the Canadian cost-effectiveness threshold. Moreover, they found that using HCICs could reduce the lifetime number of UTI events by 11%. For Japan, Watanabe et al. [80] found that an ICER of 3.8 million yen (US $ 31,405), which falls well within the Japanese societal willingness to pay per QALY gained; therefore, hydrophilic‐coated catheters can be considered highly cost‐effective in Japan when compared with uncoated catheters. Finally, Couchman et al. [56] concluded that HCICs on average help gain 0.82 life years (0.73 QALYs) per person and that the use of HCICs is broadly cost-effective in Australia.

#### Spina bifida, multiple sclerosis, and benign prostate hypertrophy

No relevant HEOR studies have been found within SB, MS, as well as any non-neurogenic diseases.

#### Mixed population

Five studies with mixed pathology within the neurogenic bladder group was identified. Shamout [6] concluded that HCICs were 4.4 times more expensive when compared with non-HCICs; however, the data was only based on a few studies, and cost savings due to possible reduction in UTI was not included in the calculation nor was any increase in QALY included. In another review by Saadat et al. [81], the authors summarized that several studies found HCICs to be more cost-effective than uncoated catheters, and the added cost of life extension is above proposed thresholds. In a recent review by Feng et al. [4], the authors concluded that despite the higher unit value, the additional cost of HCICs was offset with savings due to fewer complications in comparison with non-HCICs when considered over a lifetime and from the societal perspective. Besides this, the decrease in patient suffering from fewer complications would also add to the benefits of HCICs; this is based on an out-of-pocket model in China.

Finally, Hakansson [82] investigated cost-effectiveness of HCICs from a US perspective: At the same reimbursement level, the hydrophilic-coated catheter was found to result in both health benefits and substantial cost savings compared to the non-coated catheter. Håkansson again in 2016 concluded that the use of HCICs on average avoided 18 complications per person, which translated into a lifetime cost saving of $ 10,183 in the US and that HCIC gained 0.55 QALYS during a lifetime compared to noncoated catheters [83].

### Pain and discomfort

Pain and discomfort are subjective parameters often measured by questionnaires using a grading system or by reporting the number of patients experiencing pain or discomfort. In total, 12 articles covering pain and discomfort were found (Table 9). Several articles did not find any significant differences between HCICs and non-HCICs.

**Table 9:** Pain and discomfort

Population	Evidence Pro/con HCIC	# Original studies	# Reviews	LoE
SCI	+	1	0	2B
SB	/−	3	0	2B
MS	NA	NA	NA	NA
BPH	NA	NA	0	NA
Mixed	+ + +/−	4	4	1A-2B
**All**	**+ + +/− −**	**8**	**4**	**1A-2B**

Bold text is a summary of the total evidence level of the pathologies

*BPH* Benign prostate hypertrophy, *HCIC* Hydrophilic-coated intermittent catheters, *LoE* Level of evidence, *MS* Multiple sclerosis, *NA* Not available, *SB* Spina bifida, *SCI* Spinal cord injury

+ to + + +: The literature supports claims of hydrophilic catheters as being superior to uncoated catheters

− to − − No significant difference between hydrophilic and uncoated catheters or uncoated catheters are superior

1 and 2 refer to the (descending) level of evidence. A = systematic review/meta-analysis; B = clinical trial

#### Spinal cord injury

Only Cardenas et al. [14] investigated SCI patients with regard to discomfort. They found that hydrophilic catheters, when compared with uncoated catheters, were slightly more comfortable with regard to insertion (9.3 vs 8.9) and withdrawal (9.4 vs 9.0) on a scale ranging from 0 (severe discomfort) to 10 (no discomfort); however, the difference was not statistically significant, and whether changes that are close to “no-discomfort” on the scale are clinically relevant, is debatable.

#### Spina bifida

Three articles investigated comfort of hydrophilic versus uncoated catheters in SB patients (DeFoor et al. [19], Chick et al. [21], and Kiddoo et al. [20]). Chick et al. and Kiddoo et al. both found that the uncoated catheter was rated higher than the hydrophilic version; however, the results were not significant. In contrast, DeFoor et al. found that HCIC patients reported a decrease in discomfort with the catheterization process from 1.7 (baseline) to 0.4 (*p* = 0.06) on a scale from 0 (no discomfort) to 10 (maximum discomfort); however, four patients from the HCIC group left the study due to discomfort and/or increased bladder spasms. DeFoor recorded that three patients in the HCIC group but none in the uncoated group experienced pain (this difference was not statistically significant).

#### Multiple sclerosis and benign prostate hypertrophy

No studies on MS or BPH patients were found.

#### Mixed pathology

The EAUN guidelines from 2013 concluded that HCIC reduced pain, and most patients prefer to use HCIC for improved comfort. In patients taking a long time to catheterize, discomfort during withdrawal may occur due to drying of the hydrophilic surface [47]. In a review by Kennelly [48] comparing gel lubricated catheters to HCICs, it was concluded that HCICs cause less removal friction and pain.

Several trials concluded that HCICs significantly reduced discomfort when compared with gel lubricated (Leriche et al. [30]) or uncoated catheters (Stensballe et al. [25], Newman et al. [32]). Stensballe found that SpeediCath HCIC only caused discomfort in 30% of the users compared to 55% in the uncoated catheter group, while testing on healthy male subjects with full sensation. In the study by Leriche et al., 7/29 patients using gel-coated catheters reported pain or discomfort compared to only 1/29 using HCICs (SpeediCath) (*p* = 0.057).

The Ontario Medical Advisory Secretariat from 2006 [33] concluded that there was no difference in pain or burning sensation (based only on the study by Pachler Frimodt-Møller [22]), and that no evidence was available to indicate that HCICs are better than uncoated catheters regarding patient comfort; however, newer studies listed above disagree with this conclusion. Cindolo et al. 2004 investigated VAS scores in patients with bladder cancer. Patients using hydrophilic catheters reported significantly lower (i.e., better) VAS scores for discomfort when compared with patients using the standard catheters (1.3 vs. 2.1, *p* < 0.001 on a scale from 0 to 10).

### Other outcomes

#### Fertility

Very little documentation on the effect of HCICs on male fertility has been reported. Only one paper comparing catheter influence on sperm viability and motility is available: Auger et al. [84] tested in vitro the effect of three different catheters on sperms. No difference in sperm viability was observed. However, a significant reduction in sperm motility was observed for one HCIC (LoFric) but not another (SpeediCath); an uncoated but lubricated catheter also showed no effect on sperm motility. Further studies need to be performed before any conclusions on this topic can be made.

#### Compliance/adherence

Only a few studies have investigated the impact of catheter type on compliance/adherence. Afsar et al. [53] found no significant difference in compliance between hydrophilic and PVC catheters in SCI patients. In another study by McClurg et al. [85] in MS patients, the type of catheter did not affect the adherence to intermittent catheterization. In an opinion review by Seth et al. [86], QoL was found to be higher in patients with adherence to clean intermittent self-catheterization, and authors concluded that choosing the appropriate catheter may improve adherence. Håkansson published a review in 2014 investigating reuse versus single-use catheters and concluded that patients should be able to choose the catheter that best fits their needs and preferences, regardless of it being a more expensive choice or not, in order to ensure compliance to treatment in patients practicing intermittent catheterization [45].

#### Re-admission

No relevant documentation related to HCIC versus uncoated catheters was found.

#### Bladder stones

Evidence concerning the formation of bladder and kidney stones was sparse, possibly due to the rarity of the event. Watanabe et al. [80] and Welk et al. [79] both used a 10% reduction in the risk of stones when using hydrophilic-coated catheters compared to uncoated catheters for the calculation of cost-effectiveness. Watanabe used expert panel as reference. Vapnek et al. [27] compared 30 patients on HCIC and 31 using uncoated PVC catheters; in the uncoated catheter group, one patient reported a bladder stone.

## Discussion

UTI is a major cause of morbidity in individuals, particularly those with neurogenic bladder. Incomplete bladder emptying leading to residual urine allows growth of leftover bacteria, which may cause permanent bacteriuria. More serious consequences of UTIs include frequent recurrences, pyelonephritis, urosepsis, renal failure, and high-level antibiotic resistance [87]. UTIs are thus among the greatest risks to people undertaking intermittent catheterization. Although the risk is lower than that for indwelling catheters, on average, a clean intermittent catheter user will likely experience 2.5 UTIs/year [57, 88], with over 80% of individuals experiencing at least one UTI over a 5 year period [89]. While many studies investigate UTI’s related to intermittent catheterization and the majority of studies support hydrophilic catheters for reduction of UTI’s, conflicting evidence was found and larger more controlled studies are required.

### Summary of main results

This scoping review found 77 articles comparing hydrophilic versus non-hydrophilic intermittent catheters in terms of various outcomes and pathologies. In general, there were more reviews (n = 40) available than actual clinical studies (n = 37). The comparison of hydrophilic versus non-hydrophilic catheters for intermittent catheterization is quite well documented in patients with mixed pathology, SCI, and to some extent SB, with high levels of evidence; however, at the time of undertaking this review, no documentation was available exclusively for MS patients. Within non-neurogenic diseases like BPH and cancer, only a few studies were available. While for some of the outcomes evaluated, the conclusions from the different sources were highly in accordance with each other, for others such as UTI and adverse events, contradictory findings were found. Overall, however, the available evidence predominantly indicates better outcomes with hydrophilic catheters, particularly, greater UTI reduction and improved satisfaction, cost-effectiveness, and quality of life are reported by study authors.

With respect to UTI in HCIC and non-HCIC users, this review found contradicting results; nevertheless, more than half of the original articles (12 out of 20) and reviews (17 out of 32) concluded that HCICs help reduce UTI when compared with non-HCICs. Only five clinical studies and seven reviews concluded no difference or favored non-HCICs in this regard. In total, 10 out of the 12 articles with a level of evidence 1A or 1B favored HCICs. In children, three studies investigated UTI in relation to HCICs versus non-HCICs (Burki [11], DeFoor [19] and Kiddoo [20]). The largest study (Burki) found a significant advantage of HCICs whereas the two other studies arrived at an opposite conclusion; however, they also mention that the children found the hydrophilic catheter too slippery, something that has also been reported by Boucher [34], indicating that a clean procedure was not being applied by the children, which may very well have affected the outcome of the studies. Better training of children or better designed catheters may be needed to ensure that children also benefit from hydrophilic catheters.

Another of the key outcomes where hydrophilic catheters are speculated to make a difference is in relation to trauma and microhematuria. The hydrophilic technology adds a smooth wetted surface to the catheter, and the ability to impose lower friction force during catheterization reduces the risk of microstrictures. As reducing adverse events is one of the primary reasons for using HCICs, adverse events in relation to HCICs is well documented. There are various ways in which adverse events are defined in the literature; most articles use hematuria, although no strict definition is made except for various amounts of red blood cells in the urine, which may also be affected by the amount of urine collected and the accuracy of the measurement method (dipstick or other). Other articles use signs of blood in the urine, urethral inflammatory response [e.g., leukocyte counts (pyuria)], or long-term urethral strictures. In this review, a total of 12 clinical studies were found that discussed adverse events with more or less a 50:50 split on conclusion as to whether HCICs reduce adverse events when compared with non-HCICs or if there is no difference between the two types of devices. Four of the seven negative/non-significant studies with regard to HCICs were within SB. There was no relation between the outcome and if the non-coated catheter was lubricated or not.

On the contrary, most of the included articles agreed that hydrophilic catheters offer reduced pain and discomfort; however, in SB patients these conclusions are unclear.

The majority of articles assessing satisfaction supported higher satisfaction with HCICs over non-HCICs, and in general, most studies agreed that HCICs provide better satisfaction (Shamout [6], Hedlund [28], Salameh [18], Selius [1]). Feng [4] concluded that adults are more satisfied with HCICs (OR = 1.48); however, children prefer non-HCICs (OR = 0.39), which is in agreement with the results on patients with SCI (adults) versus SB (children). In some studies, the satisfaction level of HCICs was negatively affected by either price or initial difficulties in handling; nevertheless, in general, the available evidence supports HCIC for increased satisfaction.

Overall, all but one study found an increased preference for HCICs over other catheters; however, none of the studies had a level of evidence above 2 on the Oxford scale. Only one review (Shamout [6]) reported on preference, while indicating a low level of evidence. The review concluded that preference was significantly higher for HCICs versus uncoated catheters. Only one article out of 16 (Kiddoo et al. [20]) did not observe a preference for the HCICs; however, the study was conducted in children who often find the hydrophilic catheter to be slippery.

QoL in patients using intermittent catheterization is affected by several factors related to health such as pain, discomfort, fear of UTI, and fear of trauma; however, there are also a number of social factors affecting QoL, including willingness to go out, self-image, time to catheterize, device discreteness, embarrassment, and sexuality. When comparing the literature on the effect of HCIC on QoL, it is clear that all studies are in agreement that HCICs provide improved QoL when compared with non-coated catheters (Sutherland [39], Shamout [6], Chartier-Kastler [31]). Similarly, most research points toward hydrophilic-coated catheters being a cost-effective option over single-use non-coated catheters in multiple markets, including out-of-pocket payment markets, with substantial cost savings when compared with non-coated catheters. However, two reviews (Shamout [6], Bermingham [58]) with a high level of evidence concluded otherwise, one of them, however, on a poorly documented background (Shamout [6]).

### Comparison with other reviews

In a recent Cochrane review by Prieto et al. [90], i.e. an update of the retracted review from 2017, the authors investigated the impact of HCIC versus non-HCIC catheters on the risk of UTIs. Although they found an 11% reduced risk of getting UTIs when using hydrophilic catheters, the finding was not significant. They included a large amount of exclusion criteria and ended up with only two studies as the foundation for their conclusion. Five other systematic reviews published within the last decade [4, 5, 7, 8, 60] all found significant and larger differences in favor of HCICs. In a recent meta-analysis by Ye et al. [7] who conducted a network analysis comparing different catheters for intermittent catheterization the authors made an interesting finding which may help explain the discrepancy in the effect of hydrophilic catheters on the risk of UTI. The authors concluded that ready-to-use catheters, and in general, catheters requiring a no-touch technique showed significant advantages compared to those based on traditional techniques such as hydrophilic catheters requiring pre-activation in water before usage, as well as uncoated single and multiple-use catheters requiring lubrication and handling.

With regard to adverse events, the included studies were not in agreement whether hydrophilic coating reduced adverse event. Contrarily, only one out of 20 reviews and guidelines concluded that HCICs did not reduce the risk of adverse events (Rognoni [5]). All the other reviews favored HCICs for reduction of adverse events (Feng [4], Shamout [6], Kennelly [48]), European Association of Urology Nurses (EAUN) guidelines [47], and International Consultation on Incontinence consensus statement, Incontinence, 6th Edition [29]).

The Cochrane review by Prieto from 2017 [66] showed results significantly favoring HCICs for increased satisfaction; however, it concluded that uncoated catheters are as good as HCICs. This was later criticized by Christison et al. [60], which lead to a withdrawal of the Cochrane review (2017). Finally, Chartier-Kastler et al. [31] concluded that the high rates of satisfaction seen with HCICs compared to PVC catheters are crucial to ensure long-term compliance.

### Strengths and limitations of this review

While the search strategy for this scoping review did not include MeSH terms, it did include wildcards and found many articles that have been overlooked by other reviews. Single-author full-text screening was used to select studies and although OCEBM levels of evidence were used to grade the studies, no formal assessment of any risk of bias was made and the reported efficacy is as reported by the study authors. That no protocol was enlisted in a registry may also be considered a limitation of this study.

Heterogeneity in the results for some of the outcomes made it challenging for generating the evidence overview. Several other limitations also made data comparison difficult. For example, the time span of the studies varied from two weeks to years; however, most studies were done in weeks, which makes proper evaluation of relatively “rare” outcomes like UTI very uncertain. In terms of study size, many studies had less than 50 patients; in combination with a short study period, the strength of the results may be considered low. As such, more long-term studies involving larger population sizes should be done to assess the long-term effects of HCIC versus non-HCIC. In many studies, the control group was poorly described; especially in retrospective studies, the description of the catheters was poor, with regard to shape, lubrication, and potential reuse, which may affect several of the outcomes. Often, the control group consisted of a mix of several different catheters. In many studies, the use of prophylactic antibiotics was not considered a disqualifying factor for study participation, which poses a major problem when studying the frequency of UTI. Furthermore, several studies were severely underpowered and yet made conclusions based on the results obtained. Despite these limitations, this review comprehensively summarized a large body of evidence and included information on most relevant pathologies and outcomes. It can form a backbone for further research in this area, including focused systematic reviews.

## Conclusion

This review found that study authors conclusions generally support hydrophilic-coated catheters over non-hydrophilic catheters for most outcomes. Hydrophilic-coated catheters have been endorsed by the majority of researchers based on strong evidence supporting improved satisfaction, preference, and QoL, and also as a means to reduce UTI frequency and adverse events. Nonetheless, some researchers, who do not observe an effect on UTI and adverse events, suggest that uncoated or even reused uncoated catheters might just as well be recommended, suggesting higher quality evidence is needed. As such, longer term studies involving larger population sizes are needed to support the general finding in this review that HCICs are the preferred choice in most populations. Additional training in the pediatric population or redesigned catheters may be necessary for this group to fully benefit from the advantages of hydrophilic-coated catheters.

## Supplementary Information

**Additional file 1:** PICO tables of included studies for each outcome.

**Additional file 2:** Publications excluded from the review on full text level.

## Data Availability

All data generated or analyzed during this study are included in this published article [and its additional files].

## Abbreviations

AU: Australia
BPH: Benign prostate hypertrophy
CEBM: Oxford centre for evidence based medicine
CFU: Colony forming unit
EAUN: European association of urology nurses
EAU: European association of urology
HC: Hydrophilic-coated
HCIC: Hydrophilic-coated intermittent catheterization
HEOR: Health economics outcomes research
IC: Intermittent catheterization
ICER: Incremental cost-effectiveness ratio
ISC-Q: Intermittent self-catheterization questionnaire
LoE: Level of evidence
MS: Multiple sclerosis
NA: Not available
NICE: National institute for health and care excellence
OR: Odds ratio
QALY: Quality-adjusted life year
QoL: Quality of life
PICO: Population, intervention, comparison, and outcomes
PVC: Polyvinyl chloride
RCT: Randomized controlled trial
RR: Risk ratio
SB: Spina bifida
SCI: Spinal cord injured
UC: Uncoated
US: United States
UTI: Urinary tract infection
VAS: Visual analog scale
