# Supplementary material for: A scoping review on the impact of hydrophilic versus non-hydrophilic intermittent catheters on UTI, QoL, satisfaction, preference, and other outcomes in neurogenic and non-neurogenic patients suffering from urinary retention
Source: BMC Urol. 2022 Sep 19;22:153. doi: 10.1186/s12894-022-01102-8 (PMC9487088; doi:10.1186/s12894-022-01102-8)
Supplement: Supplementary file 1 — Additional file 1: PICO tables of included studies for each outcome. [file 12894_2022_1102_MOESM1_ESM.docx]

**Appendix 1:** **PICO tables for each outcome**

**Table S1: PICO**

| **P** | Patient, Population, or Problem | Patients with neurogenic or non-neurogenic urinary retention. P: Completed/Enrolled |
| --- | --- | --- |
| **I** | Intervention | Hydrophilic-coated intermittent catheters (HCICs) |
| **C** | Comparison or existing intervention (if appropriate) | Non hydrophilic (uncoated) catheters and (pre-)lubricated catheters |
| **O** | Outcome you would like to measure or achieve | with respect to satisfaction, preference, adverse events, urinary tract infection (UTI), quality of life (QoL), cost effectiveness, pain, and discomfort |

In the following table, color coding of concluding remarks on the different outcomes:

Green: Result significantly supports hydrophilic coated catheters

Black: Inconclusive

Orange: Results either support non-hydrophilic catheters or don’t find significant advantage of hydrophilic catheters

**Table S2: Satisfaction**

| **Reference** | **Design/Evidence** | **PICO** | **Conclusion** |
| --- | --- | --- | --- |
| **High level of Evidence** | | | |
| Feng 2020 (4) | Systemic review  1A | O: Risk of UTI/Urethral trauma/patient satisfaction/cost effectiveness | Adults are more satisfied with HCIC (OR=1,48), but children prefer non-HCIC (OR=0,39) |
| Shamout, 2017 (6) | Systemic review  2A | P: 2188  I/C: HCIC vs UC  O: UTI, urethral trauma, cost-effectiveness, quality of life and patient satisfaction | Seven articles out of 9 reported a significant higher satisfaction and/or preference reported with hydrophilic catheter, when compared with non-hydrophilic catheters. However, 2 articles reported a nonsignificant difference in overall satisfaction |
| Consensus statement 2017 (29) | Guideline on incontinence  1A |  | HCIC is associated with a better overall satisfaction |
| **Medium level of evidence** | | | |
| De-Ridder 2005 (24) | RCT, 12 months follow up  Level 2B | P: 57/123 SCI males  I/C: HCIC (SpeediCath) vs UC (Conveen)  O: UTI, time to first UTI, bleeding episodes and hematuria and satisfaction. | More patients in the SpeediCath group were very satisfied after 6 months compared to patients in the PVC group (Table 4). Although there was no significant difference, more patients/care providers in the SpeediCath group found the overall catheterization  procedure, the introduction, and withdrawal of the  catheter very easy or easy compared to the PVC group. |
| Stensballe 2005 (25) | Prospective randomized single blinded crossover study  2B | P: 40/49 healthy males  I/C: HCIC (SpeediCath) vs UC  O: Pain, friction force, hematuria, preference | SpeediCath exerted significantly lower withdrawal friction force compared to PVC and LoFric.  Hydrophilic catheters caused less pain than gel lubricated PVC. |
| Cardenas 2011 (14) | RCT, 6 months follow up,  Level 2B (high drop-out) | P: 114/224 adult new SCI male/female  I/C: HCIC (SpeediCath) vs UC (Conveen)  O: time to UTI, UTI incidence, hematuria, bleeding | The overall satisfaction reported by the participants and/or caregivers by using hydrophilic-coated catheters was significantly higher (P=0 .007) compared with the evaluation given by participants and caregivers using the uncoated catheter. |
| Cindolo 2004 (23) | RCT, 6 months follow up, 2B (no sample size rationale) | P: 83/100, bladder cancer  I/C: HCIC (EasiCath) vs UC (Nelaton PVC)  O: UTI, leukocyturia and patient satisfaction (VAS score) | HCIC users showed significantly more comfort than non-coated (p<0,001) |
| DeFoor 2017 (19) | RCT, 12 months follow up  2B | P: 55 /78 Spina Bifida, children  I/C: HCIC vs lubricated UC  O: UTI etc. | HCIC patients reported a decrease in discomfort with the catheterization process from baseline (1.7) to study end (0.4). |
| Chick 2013 (21) | Questionnaire based RCT, 6+6-month crossover  2B | P: 20/51 spina bifida children  I/C: HCIC vs UC (reused 1-2 days)  O: Adaption, ease of use self-reliance | The multiuse PVC catheter was rated as highly as or higher than the single-use hydrophilic product on all 5 questions addressing convenience, comfort, handling, satisfaction and whether they would continue to use that particular product |
| Kiddoo 2015 (20) | RCT, 2x24 weeks cross-over study  2B | P: 46/66 spina bifida children  I/C: SpeediCath vs PVC (reused min 5 times)  O: UTI, hematuria, visits to physician, satisfaction | No statistically significant differences were found  in convenience or comfort as measured by the satisfaction questionnaire. However, there was a  significant difference for ease of handling, with 40% of participants disliking the hydrophilic product, compared to 10% disliking the PVC product. If the slipperiness of the catheter was overcome, the families were pleased with it. |
| Prieto 2014  RETRACTED (26) | Systemic review (Cochrane)  2A – Due to multiple errors | O: UTI, trauma, satisfaction etc. | Several studies support improved overall satisfaction with HCIC however some has problems with attrition (Cardenas 2011 has high drop-out in the HCIC arm, which may be due to low satisfaction). |
| Vapnek 2003 (27) | RCT, 12 months follow up  Level 2B | P: 49/62 adult male IC users  I/C: HCIC (LoFric) vs UC (re-use)  O: Hematuria, UTI | Patients reported high degrees of satisfaction with the hydrophilic coated catheter and many continued to use them after the conclusion of the study |
| Lindehall 2004 (12) | Observational cohort study, 10 years  2D | P: 28 male SB patients  I/C: HCIC vs UC  O: Urethral lesions | Difficulties inserting the catheter was the same with both HCIC and uncoated |
| Hedlund 2001 (28) | Opinion review  2A | O: Urethral irritation, satisfaction, preference, bacteriuria | HCIC provides better patient satisfaction (fact) |
| Leriche 2006 (30) | Multicenter, randomized cross-over  2B | P: 29/31 male IC users  I/C: HCIC (SpeediCath) vs lubricated UC | SpeediCath® demonstrated its superiority against Actreen® in terms of ease of introduction (p = 0.0065), ease of emptying (p = 0.0157), quality of the lubricant (p <0.0001), urethral tolerance and survey performance in bed (p = 0.0157) |
| Salameh 2015 (18) | Systemic review  2A | P: SCI  O: UTI, trauma, satisfaction etc. | The use of hydrophilic-coated catheters is an innovative technique that leads to better patient comfort and satisfaction |
| Selius 2008 (1) | Systemic review 2A | O: Satisfaction, UTI, hematuria | Low-friction, hydrophilic-coated catheters increased patient satisfaction |
| Chartier-Kastler 2011 (31) | Systemic review 2A | O: UTI, microhematuria, satisfaction and QoL | High rates of satisfaction seen with HCIC compared to PVC (important to ensure long term compliance). |
| Ontario Health Technology Assessment Series 2006 (33) | Guideline on hydrophilic catheters  2A | O: UTI, QoL etc. | Insufficient evidence to indicate that hydrophilic catheters are associated with improved patient satisfaction |
| Yoshida 2018 (9) | RCT, 6 months follow up  2B | P: 29/36 Experienced female neurogenic patients  I/C: Compact HCIC (SpeediCath) vs standard uncoated (Safeed)  O: UTI and QoL | The hydrophilic catheter was considered significantly safer than the uncoated catheter and satisfaction for the hydrophilic catheter was significantly higher (13/16 vs 3/13, P=0.003) |
| **Low level of evidence** | | | |
| Sarica 2010 (17) | RCT, crossover, 0, 6, 12 and 18 weeks follow up  Level 3B (no sample size rationale) | P: 10/25, SCI male  I/C: HCIC (Rüsch) vs gel lubricated (Rüsch) vs PVC  O: UTI, micro trauma and patient satisfaction | Gel-lubricated catheter was superior to both PVC and HCIC in terms of patient satisfaction (n=10, duration 6 weeks) |
| Burki 2019 (10) | Observational cohort  3D | P: 101/131 spina bifida children  I/C: HCIC vs UC  O: UTI/complications/ satisfaction/preference | Satisfaction was 10 for HCIC and 8 for uncoated (scale 1-10), but it was not significant (p=0,63) |
| Spinu 2012 (11) | Observation case control, Questionnaire, 3D | P: 45 SCI patients with >12M experience  I/C: HCIC vs UC  O: Adaption, ease of use self-reliance | Patients that used exclusively HCIC vs. Non-HCIC expressed a significant higher satisfaction level (p-value<0.0001) |
| Wyndaele 2000 (15) | Prospective study, 1 month testing of new HCIC catheter  3D | P: 35/39 male SCI  I/C: HCIC (Urocath-Gel) vs gel coated PVC (mixed use)  O: Satisfaction and UTI | Satisfaction was better with HCIC, despite elevated costs (catheters not reimbursed in Belgium in 2000) |
| Johansen 2007 (16) | Multicenter, 2-week follow up  3E | P: 378/409 SCI  I/C: HCIC (LoFric and SpeediCath) vs pre-lubricated UC vs UC  O: Satisfaction and preference | 50% wanted to continue with HCIC and reported increased general satisfaction. hydrophilic catheters should be regarded as the gold standard for patient satisfaction. |
| Yamanishi 2017 (35) | RCT, 2x4 week cross-over study  3B | P: 32/42 male w. neurogenic bladder  I/C: HCIC (SpeediCath Compact M) vs reusable or standard non-coated Nelaton  O: Ease of use, convenience, discreteness, psychological wellbeing | No statistical difference was demonstrated in terms of ease of use, convenience, discreetness, psychological well‐being between the two-catheter use. However, there were significant differences in favor of SpeediCath in terms of the individual questionnaire: "Storage of catheters at home is inconvenient" in convenience, and "My catheter allows me to feel confident when away from home" in discreetness |
| Newman 2018 and 2020 (36, 32) | Prospective, multi-center, clinical trial  3E | P: 36/39 (18 (AU) + 21 (US)) non-neurogenic + neurogenic  I/C: HCIC vs existing re-use catheter  O: QoL (ISC-Q), satisfaction, comfort, and preference | Catheter satisfaction revealed significant differences in favor of single-use HC catheters when compared to reuse catheters. |
| Boucher 2010 (34) | Comparative prospective study  3E | P: 30/39 children (SB/SCI) mixed gender.  I/C: SpeediCath Compact vs current uncoated catheter  O: Satisfaction, preference | 50% of participants thought the HC was too slippery. |

**Table S3: Preference**

| **Reference** | **Design/Evidence** | **PICO** | **Conclusion** |
| --- | --- | --- | --- |
| **High level of Evidence** | | | |
| Shamout 2017 (6) | Systemic review  2A | P: 2188  I/C: HCIC vs UC  O: UTI, urethral trauma, cost-effectiveness, quality of life and patient satisfaction | Preference is significantly higher with HCIC vs uncoated |
| **Medium level of evidence** | | | |
| Defoor 2017 (19) | RCT, 12 m follow up  2B (high drop-out) | P: 55 /78 Spina Bifida, children  I/C: HCIC vs lubricated UC  O: UTI etc. | Most requested to continue with the hydrophilic catheter. This was reflected on the questionnaire with a rating scale of 0 for continuing the hydrophilic catheter to 10 for returning to the uncoated catheter. The HCIC patients were a mean 5.3 at baseline and 2.5 at study end (p = 0.04). |
| Lucas 2016 (38) | RCT, 12 months follow up  2B (no sample size rationale) | P: 47/50 patients with myelomeningocele, age 1-42y  I/C: HCIC vs their std UC  O: Count pathogenic bacteria | 36% continued using HCIC after the trial |
| Chick 2013 (21) | Questionnaire based RCT, 6+6-month crossover  2B | P: 20/51 spina bifida children  I/C: HCIC vs UC (reused 1-2 days)  O: Adaption, ease of use self-reliance | Adaption to HCIC was difficult, but those who got it to work, preferred HCIC (based on interview) |
| Leriche 2006 (30) | Multicenter, randomized cross-over  2B | P: 29/31 male IC users  I/C: HCIC (SpeediCath) vs lubricated UC | The patients’ global assessment was clearly in favor of HCIC Set (7.0 +/- 2.3 versus 5.7 +/- 2.5; p-value = 0.0156) and 65.5% of patients preferred to use HCIC Set in the future. |
| Johansen 2007 (16) | Observational multi center, 12 days follow up  2E | P: 378/409 adult SCI, SB male/female  I/C: HCIC vs UC  O: Preference and QoL questions | 74% of individuals using conventional PVC UC wanted to continue with a HCIC after 2 weeks of evaluation (p-value < 0.05). |
| Hedlund 2001 (28) | Opinion review, multicenter  2A | I/C: HCIC vs UC  O: Urethral irritation, satisfaction, preference, bacteriuria | There is a preference for the low friction catheter (HCIC) |
| Pinder 2015 (37) | Multicenter  2E | P: 283 SCI  I/C: HCIC vs lubricated UC | Preference for HCIC is 1.808-2.041 higher compared to lubricated catheters. Willingness to pay for HCIC is 46-79€/month vs lubricated single use catheters |
| Stensballe 2005 (25) | Prospective randomized single blinded crossover study  2B | P: 40/49 healthy males  I/C: HCIC (SpeediCath/ LoFric) vs UC  O: Pain, friction force, hematuria, preference | 93% preferred HCIC (p-value < 0.0001). |
| Vapnek 2003 (27) | RCT, 3+3+3+3 months follow up (cross-over study), Multicenter  Level 2B | P: 49/62 adult male IC users  I/C: HCIC (LoFric) vs UC (re-use)  O: Hematuria, UTI | Patients preferred the hydrophilic coated catheter because of ease of use |
| Kiddoo 2015 (20) | RCT, 2x24 weeks cross-over study  2B | P: 46/66 spina bifida children  I/C: SpeediCath vs PVC (reused min 5 times)  O: UTI, hematuria, visits to physician, satisfaction | Fewer participants in the HC group (26, 57%) answered “Yes” to “Would you continue using the product?” compared to the PVC catheter group (41, 92%). |
| Yoshida 2018 (9) | RCT, 6 months follow up  2B | P: 29/36 Experienced female neurogenic patients  I/C: Compact HCIC (SpeediCath) vs standard uncoated (Safeed)  O: UTI and QoL | The willingness (preference) to use the hydrophilic catheter was significantly higher compared to the uncoated catheter (15/16 vs 6/13, P=0.01) |
| **Low level of evidence** | | | |
| Burki 2019 (10) | Observational cohort  3D | P: 101/131 spina bifida children  I/C: HCIC vs UC  O: UTI/complications/ satisfaction/preference | 18.8% of uncoated users want to switch to HCIC and 0% want to switch the other way (p<0,005). 52.8% of uncoated users prefer HCIC and 100% of HCIC users have a preference for HCIC (p<0,0001) |
| Newman 2018 and 2020 (36, 32) | Prospective, multi-center, clinical trial  3E | P: 36/39 (18 (AU) + 21 (US)) non-neurogenic + neurogenic  I/C: HCIC vs existing re-use catheter  O: QoL (ISC-Q), satisfaction, comfort, and preference | At the end of the study, 83% of the participants preferred to continue with the single use HC catheter |
| Boucher 2010 (34) | Comparative prospective study  3E | P: 30/39 children (SB/SCI) mixed gender.  I/C: SpeediCath Compact vs current uncoated catheter  O: Satisfaction, preference | After a short trial period, most children preferred their uncoated catheter and would not change for the HC. Only 10 patients (33%) would be ready to change their actual catheter for the HC. All the females in the study who had tried the SpeediCath Compact (8/8) would be ready to switch to it from their original catheter |

**Table S4: Adverse events**

| **Reference** | **Design/Evidence** | **PICO** | **Conclusion** |
| --- | --- | --- | --- |
| **High level of Evidence** | | | |
| Rognoni 2017 (5) | Meta-analysis  1A | P: SCI | Authors did not find any risk reduction for hematuria associated to hydrophilic coated catheters |
| Feng 2020 (4) | Systemic review  1A | O: Risk of UTI/Urethral trauma/patient satisfaction/cost effectiveness | Risk of urethral damage was reduced by 55% |
| Shamout 2017 (6) | Systemic review  2A | P: 2188  I/C: HCIC vs UC  O: UTI, urethral trauma, cost-effectiveness, quality of life and patient satisfaction | HCIC significantly reduces microhematuria and the number of epithelial cell count |
| EAUN guidelines 2013 (47) | Guideline on IC in adults  1A |  | Hydrophilic coating significantly reduces the risk of microscopic hematuria in people with neurogenic dysfunction |
| Consensus Statement Incontinence 6^th^ ed. 2017 (29) | Guideline/ review  1A |  | HCIC reduce microscopic hematuria |
| Kennelly 2019 (48) | Systemic review  1A | I/C: HCIC vs UC  O: UTI, hematuria | Catheters with hydrophilic coatings caused significantly less urethral trauma (hematuria) |
| **Medium Level of Evidence** | | | |
| Stensballe 2005 (25) | Prospective randomized single blinded crossover study  2B | P: 40/49 healthy males  I/C: HCIC (SpeediCath) vs UC  O: Pain, friction force, hematuria, preference | Hydrophilic catheters caused less hematuria, less pain than gel lubricated PVC.  SpeediCath exerted significantly lower withdrawal friction force compared to 1. PVC and 2. LoFric. |
| DeFoor 2017 (19) | RCT, 12 m follow up  2B | P: 55 /78 Spina Bifida, children  I/C: HCIC vs lubricated UC  O: UTI etc. | No difference in hematuria |
| Håkansson 2014 (45) | Systemic review  2A | I/C: HCIC, vs uncoated vs re-use | Review concludes that literature supports the use of single-use hydrophilic catheters to reduce risk of urethral trauma |
| Vapnek 2003 (27) | RCT, 12 months follow up  2B | P: 49/62 adult male IC users  I/C: HCIC (LoFric) vs UC (re-use)  O: Hematuria, UTI | Significantly lower microscopic hematuria in HCIC vs PVC re-use |
| De-Ridder 2005 (24) | RCT, 12 months follow up  2B | P: 57/123 SCI males  I/C: HCIC (SpeediCath) vs UC (Conveen)  O: UTI, time to first UTI, bleeding episodes and hematuria and satisfaction. | No significant difference in bleeding episodes (69% in HCIC group vs 54.2% in UCIC group) or in micro-hematuria. |
| Cardenas 2011 (14) | RCT, 6 months follow up  2B (high drop-out)  2B | P: 114/224 adult new SCI male/female  I/C: HCIC (SpeediCath) vs UC (Conveen)  O: time to UTI, UTI incidence, hematuria, bleeding | Significant reduction in hematuria in the HCIC group (23%) vs group using uncoated catheters (34%).  The number of urethral bleedings in the HCIC group (14) was significantly higher than in the UC group (6) (P<0,05). |
| Li 2013 (8) | Systemic review  2A | P: 502/548 SCI patients  I/C: HCIC vs UC  O: Hematuria, UTI | Significant less hematuria in the HCIC group vs UC group, with an odds ratio of 0.53 (based on 303 patients). |
| Kiddoo 2015 (20) | RCT, 2x24 weeks cross-over study  2B | P: 46/66 spina bifida children  I/C: SpeediCath vs PVC (reused min 5 times)  O: UTI, hematuria, visits to physician, satisfaction | No significant difference in hematuria between HCIC and uncoated (reuse): 2.64-person weeks with hematuria in the HCIC group vs 3.15 person weeks in the uncoated group |
| Prieto 2014  (26) | Systemic review (Cochrane)  2A – Due to multiple errors | O: UTI, trauma, satisfaction etc. | Results favor both HCIC and UC depending on source, but only some data is significant. Authors conclude uncoated is as good as HCIC |
| Chartier-Kastler 2011 (31) | Systemic review 2A | O: UTI, microhematuria, satisfaction and QoL | Concludes that overall HCIC appear to reduce rates of microhematuria when compared to PVC |
| Lindehall 2004 (12) | Observational cohort study, 10 years  2D | P: 28 male SB patients  I/C: HCIC vs UC  O: Urethral lesions | The rate of complications was the same with both HCIC and uncoated |
| Hedlund 2001 (28) | Opinion review  2A | O: Urethral irritation, satisfaction, preference, bacteriuria | HCIC provides decreased urethral irritation (fact) and may reduce long-term urethral complications |
| AUA (American urology association) 2014 (50) | Guideline on Catheter-Associated UTI  2A | O: UTI | HCIC may be preferable to standard non-coated catheters and are associated with a lesser degree of urethral inflammatory response when compared to standard non-coated catheters |
| Samal 2011 (40) | RCT, 2 years follow up  2B | P: 53 male SCI patients  I/C: HCIC (SpeediCath) vs std PVC (B Braun AG)  O: UTI, bleeding | No difference in urethral trauma between the 2 groups of catheters, but the lesions resulting after using PVC catheter was of greater extent |
| South African guideline 2019 (42) | Guideline  2A | P: SCI patients  I/C: HCIC vs UC  O: UTI, hematuria, QOL | HCIC reduces risk of hematuria (based on a number of references) |
| Salameh 2015 (18) | Systemic review  2A | P: SCI  I/C: HCIC vs UC  O: UTI, trauma, satisfaction etc. | Fewer inflammatory episodes at the scrotal level; fewer episodes of post-, intra-, and inter-catheterization bleeding when using HCIC |
| Selius 2008 (1) | Systemic review 2A | O: Satisfaction, UTI, hematuria | Low-friction, hydrophilic-coated catheters decreased hematuria |
| Leriche 2006 (30) | Multicenter, randomized cross-over  2B | P: 29/31 male IC users  I/C: HCIC (SpeediCath) vs lubricated UC | No patients using HCIC (SpeediCath®) (0/29 events) experienced trauma, whereas in the gel coated group 5/29 experienced bleeding |
| **Low level of Evidence** | | | |
| Burki 2019 (10) | Observational cohort  3D | P: 101/131 Spina bifida children  I/C: HCIC vs UC  O: UTI/complications/ satisfaction/preference | No significant difference (p=0,32) in hematuria 7.5 % in uncoated vs 4.1% in HCIC |
| Spinu 2012 (11) | Observation case control, Questionnaire, 3D | P: 45 SCI patients with >12M experience  I/C: HCIC vs UC  O: Adaption, ease of use self-reliance | Patients that used exclusively HCIC vs non-HCIC presented a significantly lower number of post/intra/inter catheterization bleeding episodes (p-value: 0.0001 WT). |
| D’Hondt 2011 (41) | Opinion review  3A | I/C: IC/indwelling/ suprapubic | HCIC reduce microhematuria |
| Wyndaele 2002 (46) | Opinion review  3A |  | Less strictures and urethral inflammation when using HCIC vs PVC long-term |
| Australian guideline for IC 2019 (67) | Guideline  3A (poor quality, few references) | O: UTI, trauma | Hydrophilic catheters are recommended for patients with mild trauma |
| Benedetto 2011 (43) | Systemic review  3A | P: SCI  I/C: HCIC vs UC (among other)  O: Hematuria | The use of hydrophilic-coated catheters is an innovative technique that leads to better patient comfort and satisfaction |
| Sarica 2010 (17) | RCT, crossover, 0, 6, 12 and 18w follow up  3B | P: 10/25, SCI male  I/C: HCIC (Rüsch) vs gel lubricated (Rüsch) vs PVC  O: UTI, micro trauma and patient satisfaction | Gel-lubricated catheter was superior to both PVC and HCIC in terms of urethral microtrauma, pyuria. HCIC and gel lubricated caused less hematuria (n=10, duration 6 weeks!) |
| Ye (Conference abstract) 2019 (49) | Systemic review  3A | P: 851 with neurogenic bladder  I/C: HCIC vs lubricated vs other  O: UTI, bacteriuria, hematuria | 73,2% chance that HCIC was preferred option to reduce hematuria |

**Table S5: Urinary tract infection (UTI)**

| **Reference** | **Design/Evidence** | **PICO** | **Conclusion** |
| --- | --- | --- | --- |
| **High level of Evidence** | | | |
| Rognoni 2017 (5) | Meta-analysis  1A | P: SCI | Significant reduced UTI with HCIC vs single use. |
| Cardenas 2009 (52) | RCT, 12 months follow up  1B | P: 45/56 SCI adults  I/C: HCIC (LoFric) vs UC  O: Symptomatic UTI's treated with antibiotics | 70% in the control group had 1 or more UTI's and only 50% HCIC users had 1 or more UTIs (Not significant) |
| Christison 2018 (60) | Systemic review, 1A | I/C: HCIC/uncoated single/uncoated multiple use  O: UTI | Hydrophilic versus other catheters demonstrated a significantly lower incidence of UTI |
| Feng 2020 (4) | Systemic review  1A | O: Risk of UTI/Urethral trauma/patient satisfaction/cost effectiveness | Reduced risk of UTI by 54% (52% in adults and 59% in children) |
| Shamout 2017 (6) | Systemic review  2A | P: 2188  I/C: HCIC vs UC  O: UTI, urethral trauma, cost-effectiveness, quality of life and patient satisfaction | HCIC reduces risk of UTI, although based only on the results of De Ridder. |
| Bermingham 2013 (58) | Systemic review  1A | P: Male SCI  I/C: HCIC, vs gel reservoir vs non-coated  O: UTI, bacteremia, mortality, patient preference or comfort, and the number of catheters used per day or week (cost effectiveness) | No significant difference between HCIC and uncoated catheters in terms of monthly number of UTI's or antibiotic use |
| EAUN guidelines 2013 (47) | Guideline on IC in adults  1A |  | EAUN recommends single use aseptic technique is used to reduce UTIs and states that there is a high level of evidence that hydrophilic coating reduce risk of UTI |
| Non-Surgical Urologic management of neurogenic bladder after  SCI 2017 (87) | Guideline on IC  1A | P: SCI  I/C: HCIC vs UC | HCIC may potentially minimize UTIs, however conflicting evidence exists, and a lot of the evidence is sponsored by industry |
| Consensus Statement Incontinence 6th ed. 2017 (29) | Guideline/ review  1A |  | The evidence to suggest one specific catheter type (HCIC) is weak and choice of catheters and regimens should be made on an individual basis |
| EAU -Neurogenic-LUTD, 2013 (65) | Guidelines  1A | O: UTI (page 52) | Aseptic technique and sterile lubricated or hydrophilic catheters should be used to prevent UTI |
| HICPAC, prevention of CAUTI 2019 (88) | Guidelines  1A |  | Very low-quality evidence suggested a benefit of hydrophilic catheters over standard non-hydrophilic catheters in specific populations undergoing clean intermittent catheterization |
| Kennelly 2019 (48) | Systemic review  1A | I/C: HCIC vs UC  O: UTI, hematuria | Less UTIs associated with hydrophilic catheters than with uncoated (single use) |
| Afsar 2013 (53) | Retrospective review of medical notes, 5-84m duration  1E | P: 164 (60 on IC) adult SCI  I/C: HCIC vs nelaton vs indwelling vs suprapubic O: UTI | Incidence of UTI was 2 episodes per year for PVC and reduced to once a year for hydrophilic catheter users (P=0.05) |
| Tambyah 2012 (89) | Systemic review of 5 guidelines  1A | I/C: HCIC vs other  O: UTI | HICPAC promote HCIC, IDSA do not recommend, and CDC, EAU and SHEA have not discussed HCIC |
| **Medium level of Evidence** | | | |
| De-Ridder 2005 (24) | RCT, 12 m follow up  Level 2B | P: 57/123 SCI males  I/C: HCIC (SpeediCath) vs UC (Conveen)  O: UTI, time to first UTI, bleeding episodes and hematuria and satisfaction. | Significantly fewer people using HCIC experienced UTI compared to non-HCIC  Twice as many was free of UTI in the HCIC (SpeediCath) group (36%) vs non-HCIC (PVC) group (18%) after 1-year follow-up. Median UTI rate per1000 catheter days was lower (though not significantly): 5.4 in HCIC vs 8.1 in the non-HCIC group. |
| Cardenas 2011 (14) | RCT, 6 m follow up  Level 2B (high drop-out) | P: 114/224 adult new SCI male/female  I/C: HCIC (SpeediCath) vs UC (Conveen)  O: time to UTI, UTI incidence, hematuria, bleeding | Significant (33%) decrease in the daily risk of generating UTI and a 21% relative risk reduction among patients using HCIC in the institutional setting. The time to the first antibiotic-treated symptomatic UTI was significantly delayed in the HCIC group. No difference was observed in the community setting or full study setting. |
| DeFoor 2017 (19) | RCT, 12 m follow up  2B | P: 55 /78 Spina Bifida, children  I/C: HCIC vs lubricated UC  O: UTI etc. | Significant reduction of UTI rate in the HCIC group (2/22 (9.1%) UTI's/person-year) vs uncoated (17/33, 51.5%). Baselines were similar in the year prior to the study. |
| Vapnek 2003 (27) | RCT, 12 m follow up  Level 2B | P: 49/62 adult male IC users  I/C: HCIC (LoFric) vs UC (re-use)  O: Hematuria, UTI | Significantly larger drop in UTI frequency in HCIC vs PVC re-use (but from a higher starting point… end frequency was the same for both catheters) |
| Woodbury 2008 (57) | Retrospective Self-reported national survey  2D | P: 505 adult SCI patients  I/C: Various IC products  O: Symptoms of UTI, quality of life | Fewer UTI with HCIC (2.46/year) vs non-coated (2.62/year) (NB! Re-use) |
| Lucas 2016 (38) | RCT, 12 months follow up  2B | P: 47/50 patients with myelo-meningocele, age 1-42y  I/C: HCIC (Wellspect) vs their std UC  O: Count pathogenic bacteria | Same frequency of UTI, but higher number of samples positive of pathogenic bacteria in the uncoated vs HCIC (not significant) |
| Li 2013 (8) | Systemic review  2A | P: 502/548 SCI patients  I/C: HCIC vs UC  O: Hematuria, UTI | Significant lower incidence of UTI (discrepancy in interpretation of Cardenas data) |
| Cindolo 2004 (23) | RCT, 6 months follow-up,  2B | P: 83/100, bladder cancer  I/C: HCIC (EasiCath) vs UC (Nelaton PVC)  O: UTI, leukocyturia and patient satisfaction (VAS score) | HCIC was associated with a significantly lower occurrence of UTI (3.5% vs 7.4%) |
| Nicolle 2014 (59) | Opinion review  2A | P: SCI  I/C: HCIC vs UC | The influence of HCIC on the frequency of UTI's remains controversial and based on available studies they conclude that HCIC does not affect frequency of UTI |
| Kiddoo 2015 (20) | RCT, 2x24 weeks cross-over study  2B | P: 46/66 spina bifida children  I/C: SpeediCath vs PVC (reused min 5 times)  O: UTI, hematuria, visits to physician, satisfaction | No significant difference in febrile UTI, multi-use group had significantly less weeks of self-reported UTI (study was underpowered) |
| Håkansson 2014 (45) | Systemic review  2A | I/C: HCIC, vs uncoated vs re-use | Review concludes that literature supports the use of single-use hydrophilic catheters to reduce risk of urethral trauma and urinary tract infection |
| Prieto 2014 (26) | Systemic review (Cochrane)  2A – Due to multiple errors | O: UTI, trauma, satisfaction etc. | Results favor HCIC, but only some data is significant. Authors conclude uncoated is as good as HCIC |
| Ercole 2013 (68) | Opinion review  2A | I/C: HCIC vs plastic vs PVC | HCIC compared to plastic, reduced UTI in self-catheterization; HCIC, when compared to PVC, presented a lower rate of UTI, but this association was not significant |
| Chartier-Kastler 2011 (31) | Systemic review 2A | O: UTI, microhematuria, satisfaction and QoL | Data on UTI and bacteriuria are complicated. Reduced rates are seen with HCIC compared to PVC in SCI, but few data are available within MS and SB |
| Massa 2009 (54) | RCT, 3 months follow-up  2B | P: 51/56 adult SCI patients  I/C: HCIC vs UC  O: UTI, self-diagnosis | Higher frequency of UTI's in the HCIC arm, but not significant |
| Moore 2007 (66) | Systemic review (Cochrane)  2A |  | There is a lack of evidence to state that incidence of UTI is affected by use of sterile or clean technique, coated or uncoated catheters |
| Ontario Health Technology Assessment Series 2006 (33) | Guideline on hydrophilic catheters  2A | O: UTI, QoL etc. | Insufficient evidence to indicate that hydrophilic catheters are associated with a lower rate of UTIs |
| Hedlund 2001 (28) | Opinion review  2A | O: Urethral irritation, satisfaction, preference, bacteriuria | Data suggest that HCIC may lead to a decrease in bacteriuria |
| Samal 2011 (40) | RCT, 2 years follow up  2B | P: 53 male SCI patients  I/C: HCIC (SpeediCath) vs std PVC (B Braun AG)  O: UTI, bleeding | Significantly lower incidence of symptomatic urinary tract infections in the group using hydrophilic catheters, 0.94 vs 1.58 (p < 0.05) |
| Romo 2018 (62) | Systemic review  2A | P: SCI patients  I/C: HCIC vs UC | limited evidence suggesting that the use of lubricated hydrophilic catheters is associated with lower risk of symptomatic urinary infections in SCI patients |
| South African guideline (42) | Guideline  2A | P: SCI patients  I/C: HCIC vs UC  O: UTI, hematuria, QOL | HCIC reduces risk of UTI (based on a number of references) |
| Salameh 2015 (18) | Systemic review  2A | P: SCI  I/C: HCIC vs UC  O: UTI, trauma, satisfaction etc. | Hydrophilic-coated catheters have been associated with lower rates of symptomatic urinary tract infection in patients with acute spinal cord injury |
| Selius 2008 (1) | Systemic review 2A | O: Satisfaction, UTI, hematuria | Low-friction, hydrophilic-coated catheters decreased urinary tract infection |
| Madersbacher 2017 (70) | Systemic review  2A | I/C: HCIC vs lubricated vs other | Coated catheters have yielded better results in a series of studies than the uncoated catheters, but clear evidence is not present due to the design of the studies undertaken |
| Yoshida 2018 (9) | RCT, 6 months follow up  2B | P: 29/36 Experienced female neurogenic patients  I/C: Compact HCIC (SpeediCath) vs standard uncoated (Safeed)  O: UTI and QoL | The incidence of antibiotic-treated symptomatic UTIs (n=2, 12,5%) in the HC group tended to be lower than that (n=5, 38,5%) in the UC group, but the difference was not statistically significant (p=0.192) |
| **Low level of Evidence** | | | |
| Burki 2019 (10) | Observational cohort  3D | P: 101/131 Spina bifida children  I/C: HCIC vs UC  O: UTI/complications/ satisfaction/preference | Fewer UTI's in the uncoated group, but not significant (p=0,09) |
| Spinu 2012 (11) | Observation case control, Questionnaire, 3D | P: 45 SCI patients with >12M experience  I/C: HCIC vs UC  O: Adaption, ease of use self-reliance | Slightly fewer UTI's in the HCIC group, but not significant |
| Sarica 2010 (17) | RCT, crossover, 0, 6, 12 and 18 weeks follow up  3B | P: 10/25, SCI male  I/C: HCIC (Rüsch) vs gel lubricated (Rüsch) vs PVC  O: UTI, micro trauma and patient satisfaction | UTI was reduced in Gel-lubricated catheter (1/10) and HCIC (1/10) vs PVC (4/10), but not significant |
| Tenke 2014 (69) | Opinion review  3A | I/C: IC, indwelling and suprapubic | HCIC reduce risk of UTI compared to uncoated (based on conclusions from Li et al 2013) |
| D’Hondt 2011 (41) | Opinion review  3A | I/C: IC/indwelling/ suprapubic | HCIC reduce UTI |
| Wyndaele 2000 (15) | Prospective study, 1 month follow up  3D | P: 35/39 adult SCI patients  I/C: HCIC (Urocath-Gel) vs gel coated PVC (15 re-used and 24 single use)  O: Satisfaction, (UTI) | No difference in UTI before or after changing to HCIC |
| Samal 2012 (55) | RCT, 3 months follow up  3B | P: 86 male SCI patients new to IC  I/C: HCIC vs standard PVC  O: UTI, bacteriuria | The use of HCIC significantly reduce the incidence of symptomatic UTI (p<0.05) |
| Australian guideline for IC, 2019 (67) | Guideline  3A (poor quality, few references) | O: UTI, trauma | There is currently no established benefit in terms of reducing risk of UTIs between catheter types |
| Hill, 2013 (61) | Systemic review  3A | P: SCI  I/C: HCIC vs UC  O: UTI | Data on UTI and HCIC is currently controversial |
| Biardeau 2016 (64) | Systemic review  3A | I/C: HCIC vs lubricated non-coated vs reuse  O: UTI | HCIC and pre-lubricated catheters significantly reduce risk of UTI and delay the onset of UTI. However, they do not seem to have a significant impact on urinary tract colonization |
| Ye (Conference abstract) 2019 (49) | Systemic review  3A | P: 851 with neurogenic bladder  I/C: HCIC vs lubricated vs other  O: UTI, bacteriuria, hematuria | Gel lubricated sterile non-coated catheters ranked best for asymptomatic bacteriuria and symptomatic UTI, OR (0.40) in favor of HCIC vs uncoated catheter |
| Couchman (Conference Abstract) 2019 (56) | Cost effectiveness model  3C | P: SCI  I/C: HCIC vs UC  O: HEOR (UTI) | HCIC reduce total number of UTIs with 4 events per lifetime per patient |
| Neovius (Conference abstract) 2015 (71) | Questionnaire, retrospective study  3D | I/C: HCIC (Wellspect) vs their normal uncoated  O: UTI | Switching to HCIC reduces infections/UTI |

**Table S6: Quality of life (QoL)**

| **Reference** | **Design/Evidence** | **PICO** | **Conclusion** |
| --- | --- | --- | --- |
| **High level of Evidence** | | | |
| Shamout 2017 (6) | Systemic review  2A | P: 2188  I/C: HCIC vs UC  O: UTI, urethral trauma, cost-effectiveness, quality of life and patient satisfaction | Evidence supports the benefits of hydrophilic coated catheters in terms of quality of life: 7 out of 9 papers report significant improvement in satisfaction and preference with HCIC vs uncoated and the 2 last papers reported an unsignificant difference. |
| **Medium level of Evidence** | | | |
| South African guideline (42) | Guideline  2A | P: SCI patients  I/C: HCIC vs UC  O: UTI, hematuria, QOL | Single-use hydrophilic-coated catheters increase social participation by saving time, increasing independence, and reducing the burden related to IC |
| Chartier-Kastler 2011 (31) | Systemic review 2A | O: UTI, microhematuria, satisfaction and QoL | The available data indicates that hydrophilic catheters may be preferable to PVC catheters in terms of safety and quality of life |
| DeFoor 2017 (19) | RCT, 12 months follow up  2B | P: 55 /78 Spina Bifida, children  I/C: HCIC vs lubricated UC  O: UTI etc. | HCIC patients reported a decrease in discomfort with the catheterization process from baseline (1.7) to study end (0.4). All other QoL scores were unchanged.  Questionnaire not validated |
| Yoshida 2018 (9) | RCT, 6 months follow up  2B | P: 29/36 Experienced female neurogenic patients  I/C: Compact HCIC (SpeediCath) vs standard uncoated (Safeed)  O: UTI and QoL | All of the 4 subscales for Qualiveen® were significantly better in the HC group than those in the UC group, suggesting the HC group experienced better QoL. |
| **Low level of Evidence** | | | |
| D’Hondt 2011 (41) | Opinion review  3A | I/C: IC/indwelling/ suprapubic | HCIC may be preferably to PVC catheters in terms of quality of life |
| Newman 2018 and 2020 (32, 36) | Prospective, multi-center, clinical trial  3E | P: 36/39 (18 (AU) + 21 (US)) non-neurogenic + neurogenic  I/C: HCIC vs existing re-use catheter  O: QoL (ISC-Q), satisfaction, comfort, and preference | Mean ISC-Q score increased from 58.00 to 67.19 when patients switched to the single-use HC catheters. This corresponds to a statistically significant change of 9.42 units (p=0.0101) and a 20% increase in health-related quality of life |

**Table S7: Health economics and outcomes research (HEOR)**

| **Reference** | **Design/Evidence** | **PICO** | **Conclusion** |
| --- | --- | --- | --- |
| **High level of Evidence** | | | |
| Rognoni 2017 (5) | Meta-analysis  1A | P: SCI | Considered over a lifetime HCIC are a potentially cost-effective choice in comparison to uncoated ones |
| Shamout 2017 (6) | Systemic review  2A | P: 2188  I/C: HCIC vs UC  O: UTI, urethral trauma, cost-effectiveness, quality of life and patient satisfaction | HCIC is 4.4 times more expensive compared to Mentor (Pachler), provided that there is no significant difference in terms of patient’s satisfaction or complication rate (!) |
| Bermingham 2013 (58) | Systemic review  1A | P: Male SCI  I/C: HCIC, vs gel reservoir vs non-coated  O: UTI, bacteremia, mortality, patient preference or comfort, and the number of catheters used per day or week (cost effectiveness) | ICER=£54350 for pre lubricated vs uncoated (reuse) which is above limit of being cost effective. HCIC is more expensive and less effective /pre-lubricated, n=1) |
| Feng 2020 (4) | Systemic review  1A | O: Risk of UTI/Urethral trauma/patient satisfaction/cost effectiveness | HCIC is highly cost-effective compared to non-HCIC when considering over a lifetime and this cost is well within the threshold for the healthcare system. Besides, the decrease in patient suffering from fewer complications would also add to the benefits of HCIC. |
| Truzzi 2018 (75) | Cost effectiveness model  1C | P: SCI (Brazil)  I/C: HCIC vs non-HCIC  O: QALY years (Brazil) | Despite a difference in unit cost of the 2 different types of catheters, the hydrophilic coated catheters seem to be cost-effective within a lifetime perspective for SCI patients |
| **Medium level of Evidence** | | | |
| Saadat 2019 (78) | Systemic review  2A | I/C: HCIC vs multi-use  O: Risk of UTI, cost | Summarize that several studies conclude that HCIC is more cost effective than uncoated and that the added cost of life extension is above proposed thresholds |
| Håkansson 2014 (90) | Systemic review  2A | I/C: HCIC, vs uncoated vs re-use | At the same reimbursement level, the hydrophilic-coated catheter was found to result in both health benefits and substantial cost savings compared to the non-coated catheter. |
| Clark 2015 (73) | Cost-effectiveness analysis  2C | P: Adult SCI  I/C: HCIC vs UC  O: lifetime costs and quality adjusted life years | Use of HCIC in SCI patients is highly cost effective. +1.4 years and a 16% reduction in UTI's |
| Welk 2018 (76) | Cost effectiveness model  2C | P: SCI (Canada)  I/C: HCIC vs non-HCIC  O: QALY years (Canada) | The results suggest that reimbursement of HCICs should be considered in these settings (SCI in Canada). Cost-effectiveness ratio (ICER) of $66,634/QALY (=cost effective). Moreover, using HCICs could reduce the lifetime number of UTI events by 11%. |
| Watanabe 2017 (77) | Cost effectiveness model  2C | P: SCI (Japan)  I/C: HCIC vs non-HCIC  O: QALY years (Japan) | The ICER of 3.8 million yen (US$ 31 405) falls well within the Japanese societal willingness to pay per QALY gained; therefore, hydrophilic‐coated catheters can be considered highly cost‐effective in Japan compared with uncoated catheters |
| **Low level of Evidence** | | | |
| Couchman (Conference Abstract) 2019 (56) | Cost effectiveness model  3C | P: SCI (Australia)  I/C: HCIC vs UC  O: HEOR (UTI) | HCIC on average gains 0.82 life years (0.73 QALYs) per person - Use of HCIC is broadly cost-effective |

**Table S8: Pain and discomfort**

| **Reference** | **Design/Evidence** | **PICO** | **Conclusion** |
| --- | --- | --- | --- |
| **High level of Evidence** | | | |
| EAUN guidelines 2013 (47) | Guideline on IC in adults  1A |  | HCIC reduce pain and most patients prefer to use HCIC for improved comfort. In patients taking long time to catheterize, discomfort in withdrawal may occur. |
| Kennelly 2019 (48) | Systemic review  1A | I/C: HCIC vs UC  O: UTI, hematuria | Compared to gel lubricated catheters, HCIC cause less removal friction and less pain |
| **Medium Level of Evidence** | | | |
| Stensballe 2005 (25) | Prospective randomized single blinded crossover study  2B | P: 40/49 healthy males  I/C: HCIC (SpeediCath/LoFric) vs UC  O: Pain, friction force, hematuria, preference | The hydrophilic catheters performed better than the uncoated with regard to discomfort (No discomfort: SpeediCath: 70%, LoFric: 68%, uncoated: 45%) |
| DeFoor 2017 (19) | RCT, 12 months follow up  2B | P: 55 /78 Spina Bifida, children  I/C: HCIC vs lubricated UC  O: UTI etc. | 3/37 using HCIC experienced urethral pain and 0/41 using UC experienced pain (p=0.06). HCIC patients reported a decrease in discomfort with the catheterization process from 1.7 (baseline) to 0.4 (P=0.06). 4 patients from the HCIC group left the study due to discomfort and/or increased bladder spasms |
| Cardenas 2011 (14) | RCT, 6 months follow up  2B (high drop-out)  2B | P: 114/224 adult new SCI male/female  I/C: HCIC (SpeediCath) vs UC (Conveen)  O: time to UTI, UTI incidence, hematuria, bleeding | HCIC vs UC was more comfortable with regard to insertion (9.3 vs 8.9) and withdrawal (9.4 vs 9.0) but not significant |
| Kiddoo 2015 (20) | RCT, 2x24 weeks cross-over study  2B | P: 46/66 spina bifida children  I/C: SpeediCath vs PVC (reused min 5 times)  O: UTI, hematuria, visits to physician, satisfaction | No significant difference in comfort between HCIC (87.5% acceptable) and uncoated (95.7% acceptable) p-value>0.05 |
| Leriche 2006 (30) | Multicenter, randomized cross-over  2B | P: 29/31 male IC users  I/C: HCIC (SpeediCath) vs lubricated UC | No patients using HCIC (SpeediCath®) (0/29 events) experienced trauma, whereas in the gel coated group 5/29 experienced bleeding |
| Ontario Health Technology Assessment Series 2006 (33) | Guideline on hydrophilic catheters  2A | O: UTI, QoL etc. | No difference in pain or burning sensation (based only on Pachler Frimodt-Møller 1999). No evidence available to indicate that HCIC is better than uncoated regarding patient comfort |
| Cindolo 2004 (23) | RCT, 6 months follow up, 2B | P: 83/100, bladder cancer  I/C: HCIC (EasiCath) vs UC (Nelaton PVC)  O: UTI, leukocyturia and patient satisfaction (VAS score) | Patients using the hydrophilic catheters reported significantly lower VAS scores for discomfort compared with the patients using the standard catheters (1.3 vs. 2.1, P < .001) |
| Chick 2012 (21) | Questionnaire based RCT, 6+6-month crossover  2B | P: 20/51 spina bifida children  I/C: HCIC vs UC (reused 1-2 days)  O: Adaption, ease of use self-reliance | The uncoated multiuse catheter was rated higher in comfort compared to the hydrophilic catheter (not statistically significant) |
| **Low level of Evidence** | | | |
| Newman 2020 (32, 36) | Prospective, multi-center, clinical trial  3E | P: 36/39 (18 (AU) + 21 (US)) non-neurogenic + neurogenic  I/C: HCIC vs existing re-use catheter  O: QoL (ISC-Q), satisfaction, comfort, and preference | Fewer patients reported discomfort or pain with the single-use HCIC as compared to the reuse catheter (33% vs 44%, p-value 0.0192) |
