# Supplementary material for: A scoping review on the impact of hydrophilic versus non-hydrophilic intermittent catheters on UTI, QoL, satisfaction, preference, and other outcomes in neurogenic and non-neurogenic patients suffering from urinary retention
Source: BMC Urol. 2022 Sep 19;22:153. doi: 10.1186/s12894-022-01102-8 (PMC9487088; doi:10.1186/s12894-022-01102-8)
Supplement: Supplementary file 2 — Additional file 2: Publications excluded from the review on full text level. [file 12894_2022_1102_MOESM2_ESM.docx]

**Appendix 2:** **Publications excluded from the review on full text level**

**Table S1: Excluded publications**

| **Reference** | **Publication year** | **Level of exclusion** | **Reason for exclusion** |
| --- | --- | --- | --- |
| Jeong (1) | 2019 | Full text | Review with limited conclusion on HCIC |
| Tradewell (2) | 2018 | Full text | Review with limited conclusion on HCIC |
| Lamin (3) | 2016 | Full text | Review with limited conclusion on HCIC |
| Nazarko (4) | 2012 | Full text | Review with limited conclusion on HCIC |
| Denys (5) | 2012 | Full text | Compare one hydrophilic vs other hydrophilic (HCIC vs HCIC) |
| Witjes (6) | 2009 | Full text | Compare one hydrophilic vs other hydrophilic (HCIC vs HCIC) |
| Taskinen (7) | 2008 | Full text | Compare one hydrophilic vs other hydrophilic (HCIC vs HCIC) |
| Litherland (8) | 2007 | Full text | Compare one hydrophilic vs other hydrophilic (HCIC vs HCIC) |
| Getliffe (9) | 2007 | Full text | Reuse |
| Bjerklund (10) | 2007 | Full text | Duplicate, Similar to Johansen et al 2007 (11) |
| Hudson (12) | 2005 | Full text | In vitro |
| Pascoe (13) | 2001 | Full text | Compare one hydrophilic vs other hydrophilic (HCIC vs HCIC) |
| Fader (14) | 2001 | Full text | Compare one hydrophilic vs other hydrophilic (HCIC vs HCIC) |
| Lundgren (15) | 2000 | Full text | Animal study |
| Moore (16) | 2009 | Full text | Poster on partial Cochrane review |
| Giannantoni (17) | 2001 | Full text | No HCIC |
| Igawa (18) | 2008 | Full text | No HCIC |
| Newman (19) | 2011 | Full text | Review with limited conclusion on HCIC |
| Cambell (20) | 2004 | Full text | Review with limited conclusion on HCIC |
| Nicolle (21) | 2014 | Full text | No HCIC |
| Wilde (22) | 2011 | Full text | No HCIC |
| Lindehall (23) | 2007 | Full text | No HCIC |
| Trautner (24) | 2002 | Full text | No HCIC |
| AUA/SUFU (25) | 2021 | Full text | Guideline with no recommendations on HCIC |
| CUAJ-CUA (26) | 2020 | Full text | Guideline with no recommendations on HCIC |
| EAU/ESPU (27) | 2020 | Full text | Guideline with no recommendations on HCIC |
| EAU Guidelines on Neuro-Urology (28) | 2016 | Full text | Guideline with no recommendations on HCIC |
| EAU Guidelines on Neuro-Urology (29) | 2020 | Full text | Guideline with no recommendations on HCIC |
| EAU Guidelines on Urologic Infections (30) | 2018 | Full text | Guideline with no recommendations on HCIC |
| EAU Guidelines on Paediatric Urology (31) | 2016 | Full text | Guideline with no recommendations on HCIC |
| EAU Guidelines on Urinary Incontinence in Adults (32) | 2016 | Full text | Guideline with no recommendations on HCIC |
| EAU Guidelines on Urinary Incontinence in Adults (33) | 2020 | Full text | Guideline with no recommendations on HCIC |
| EAU Guidelines on Urinary Incontinence (34) | 2015 | Full text | Guideline with no recommendations on HCIC |
| HICPAC: Guideline for prevention of catheter-associated urinary tract infections (35) | 2009 | Full text | Guideline with no recommendations on HCIC |
| Guidelines for Clinical Management of Infectious Disease 2015 - Urinary tract infection/male genital infection (36) | 2017 | Full text | Guideline with no recommendations on HCIC |
| Clinical guidelines for the diagnosis and treatment of lower urinary tract dysfunction in patients with spinal cord injury (37) | 2020 | Full text | Guideline with no recommendations on HCIC |
| Guidelines for management of urinary incontinence (38) | 2008 | Full text | Guideline with no recommendations on HCIC |
| Clinical guidelines for male lower urinary tract symptoms and benign prostatic hyperplasia (39) | 2017 | Full text | Guideline with no recommendations on HCIC |
| Guidelines for Infection Control in the Urological Field, including Urinary Tract Management (revised second edition) (40) | 2021 | Full text | Guideline with no recommendations on HCIC |
| Lower urinary tract symptoms in men: management (41) | 2010 | Full text | Guideline with no recommendations on HCIC |
| Urinary incontinence: the management of urinary incontinence in women (42) | 2006 | Full text | Guideline with no recommendations on HCIC |
| Bladder Management for Adults with Spinal Cord Injury: A Clinical Practice Guideline for Health-Care Providers (43) | 2006 | Full text | Guideline with no recommendations on HCIC |
| International Perspectives on Spinal Cord Injury (44) | 2013 | Full text | Guideline with no recommendations on HCIC |
| Japanese Clinical Guideline for Female Lower Urinary Tract Symptoms (45) | 2016 | Full text | Guideline with no recommendations on HCIC |

**References:**

1. Jeong SJ, Oh SJ. Recent Updates in Urinary Catheter Products for the Neurogenic Bladder Patients with Spinal Cord Injury. Korean J Neurotrauma. 2019;15(2):77-87.

2. Tradewell M, Pariser JJ, Nimeh T, Elliott SP, Neurogenic Bladder Research G. Systematic review and practice policy statements on urinary tract infection prevention in adults with spina bifida. Transl Androl Urol. 2018;7(Suppl 2):S205-S19.

3. Lamin E, Newman DK. Clean intermittent catheterization revisited. Int Urol Nephrol. 2016;48(6):931-9.

4. Nazarko L. Intermittent self-catheterisation: past, present and future. Br J Community Nurs. 2012;17(9):408, 10-12.

5. Denys P, Previnaire JG, Aegerter P, de Seze M, Karsenty G, Amarenco G. Intermittent self-catheterization habits and opinion on aseptic VaPro catheter in French neurogenic bladder population. Spinal Cord. 2012;50(11):853-8.

6. Witjes JA, Del Popolo G, Marberger M, Jonsson O, Kaps HP, Chapple CR. A multicenter, double-blind, randomized, parallel group study comparing polyvinyl chloride and polyvinyl chloride-free catheter materials. J Urol. 2009;182(6):2794-8.

7. Taskinen S, Fagerholm R, Ruutu M. Patient experience with hydrophilic catheters used in clean intermittent catheterization. J Pediatr Urol. 2008;4(5):367-71.

8. Litherland AT, Schiotz HA. Patient-perceived discomfort with two coated urinary catheters. Br J Nurs. 2007;16(5):284-7.

9. Getliffe K, Fader, M., Allen, C., Pinar, K., Moore, K.N. Current Evidence on Intermittent Catheterization. J Wound Ostomy Continence Nurs. 2007;34(3):8.

10. Bjerklund Johansen T, Hultling C, Madersbacher H, Del Popolo G, Amarenco G. A novel product for intermittent catheterisation: its impact on compliance with daily life--international multicentre study. Eur Urol. 2007;52(1):213-20.

11. Johansen TB, Hultling C, Madersbacher H, Del Popolo G, Amarenco G, Group LPS. A novel product for intermittent catheterisation: its impact on compliance with daily life—international multicentre study. European urology. 2007;52(1):213-20.

12. Hudson E, Murahata RI. The 'no-touch' method of intermittent urinary catheter insertion: can it reduce the risk of bacteria entering the bladder? Spinal Cord. 2005;43(10):611-4.

13. Pascoe G, Clovis S. Evaluation of two coated catheters in intermittent self-catheterization. Br J Nurs. 2001;10(5):325-9.

14. Fader M, Moore KN, Cottenden AM, Pettersson L, Brooks R, Malone-Lee J. Coated catheters for intermittent catheterization: smooth or sticky? BJU Int. 2001;88(4):373-7.

15. Lundgren J, Bengtsson O, Israelsson A, Jonsson AC, Lindh AS, Utas J. The importance of osmolality for intermittent catheterization of the urethra. Spinal Cord. 2000;38(1):45-50.

16. Moore K, Fader, M., Getliffe, K. Intermittent catheterisation: do different catheter types, strategies or techniques affect UTI? Neurourology and urodynamics. 2009;28(7):2.

17. Giannantoni A, DI STASI SM, Scivoletto G, Virgili G, Dolci S, Porena M. Intermittent catheterization with a prelubricated catheter in spinal cord injured patients: A prospective randomized crossover study. The Journal of urology. 2001;166(1):130-3.

18. Igawa Y, Wyndaele JJ, Nishizawa O. Catheterization: possible complications and their prevention and treatment. Int J Urol. 2008;15(6):481-5.

19. Newman DK, Willson, M.M. Review of Intermittent Catheterization and Current Best Practicises. Urologic Nursing. 2011;31(1):20.

20. Campbell JB, Moore KN, Voaklander DC, Mix LW. Complications associated with clean intermittent catheterization in children with spina bifida. J Urol. 2004;171(6 Pt 1):2420-2.

21. Nicolle L. Catheter associated urinary tract infections. Antimicrobial Resistance and Infection Control. 2014;3(23):8.

22. Wilde MH, Brasch J, Zhang Y. A qualitative descriptive study of self-management issues in people with long-term intermittent urinary catheters. J Adv Nurs. 2011;67(6):1254-63.

23. Lindehall B, Abrahamsson K, Jodal U, Olsson I, Sillen U. Complications of clean intermittent catheterization in young females with myelomeningocele: 10 to 19 years of followup. J Urol. 2007;178(3 Pt 1):1053-5.

24. Trautner BW, Darouiche RO. Prevention of urinary tract infection in patients with spinal cord injury. J Spinal Cord Med. 2002;25(4):277-83.

25. Ginsberg DA, Boone TB, Cameron AP, Gousse A, Kaufman MR, Keays E, et al. The AUA/SUFU Guideline on Adult Neurogenic Lower Urinary Tract Dysfunction: Treatment and Follow-up. J Urol. 2021;206(5):1106-13.

26. Campeau L, Shamout S, Baverstock RJ, Carlson KV, Elterman DS, Hickling DR, et al. Canadian Urological Association Best Practice Report: Catheter use. Can Urol Assoc J. 2020;14(7):E281-E9.

27. Stein R, Bogaert G, Dogan HS, Hoen L, Kocvara R, Nijman RJM, et al. EAU/ESPU guidelines on the management of neurogenic bladder in children and adolescent part I diagnostics and conservative treatment. Neurourol Urodyn. 2020;39(1):45-57.

28. B. Blok JP, D. Castro-Diaz,, G. del Popolo JG, R. Hamid, G. Karsenty, T.M. Kessler, Guidelines Associates: R. Boissier HE, B. Padilla Fernández TG, L. ‘t Hoen, S. Musco, V. Phé,, Schneider MP. EAU Guidelines on Neuro-urology. European Association of Urology. 2016.

29. B. Blok DC-D, G Del Popolo, J. Groen, R. Hamid, G. Karsenty, T.M. Kessler, J. Pannek. EAU Guidelines Neuro-Urology. European Association of Urology. 2020:52.

30. G. Bonkat RP, R. Bartoletti, T. Cai, F. Bruyère, S.E. Geerlings, B. Köves, F. Wagenlehner. EAU Guidelines on Urological-Infections. European Association of Urology. 2018:66.

31. S. Tekgül HSD, P. Hoebeke, R. Kocvara,, J.M. Nijman CR, R. Stein. EAU Guidelines Paediatric Urology. European Association of Urology. 2016:136.

32. F.C. Burkhard MGL, L.C. Berghmans, J.L.H.R. Bosch, F. Cruz, G.E. Lemack, A.K. Nambiar, C.G. Nilsson, R. Pickard, A. Tubaro. EAU Guidelines Urinary Incontinence in Adults. European Association of Urology. 2016:88.

33. F.C. Burkhard JLHRB, F. Cruz, G.E. Lemack, A.K. Nambiar, N. Thiruchelvam, A. Tubaro. EAU Guidelines Urinary Incontinence in Adults. European Association of Urology. 2020:100.

34. M.G. Lucas DB, L.C. Berghmans JLHRB, F.C. Burkhard, F. Cruz,, A.K. Nambiar CGN, A. Tubaro, R.S. Pickard. Guidelines on Urinary Incontinence. European Association of Urology. 2015:75.

35. Gould CV, Umscheid CA, R.K. A, Kuntz G, Pegues DA. GUIDELINE FOR PREVENTION OF CATHETER-ASSOCIATED URINARY TRACT INFECTIONS. Healthcare Infection Control Practices Advisory Committee (HICPAC). 2019:1-61.

36. Japanese Association for Infectious Disease/Japanese Society of C, Committee JJGGtCMoIDP, Urinary tract infection/male genital infection working g, Yamamoto S, Ishikawa K, Hayami H, et al. JAID/JSC Guidelines for Clinical Management of Infectious Disease 2015 - Urinary tract infection/male genital infection. J Infect Chemother. 2017;23(11):733-51.

37. Sekido N, Igawa Y, Kakizaki H, Kitta T, Sengoku A, Takahashi S, et al. Clinical guidelines for the diagnosis and treatment of lower urinary tract dysfunction in patients with spinal cord injury. Int J Urol. 2020;27(4):276-88.

38. Nishizawa O, Ishizuka O, Okamura K, Gotoh M, Hasegawa T, Hirao Y. Guidelines for management of urinary incontinence. Int J Urol. 2008;15(10):857-74.

39. Homma Y, Gotoh M, Kawauchi A, Kojima Y, Masumori N, Nagai A, et al. Clinical guidelines for male lower urinary tract symptoms and benign prostatic hyperplasia. Int J Urol. 2017;24(10):716-29.

40. Takahashi S, Arakawa S, Ishikawa K, Kamei J, Kobayashi K, Shigemura K, et al. Guidelines for Infection Control in the Urological Field, including Urinary Tract Management (revised second edition). Int J Urol. 2021;28(12):1198-211.

41. NICE lower urinary tract symptoms in men: management. National Institute for Health and Care Excellence (NICE). 2010:27.

42. Urinary incontinence: the management of urinary incontinence in women. National Institute for Health and Care Excellence (NICE). 2006:28.

43. T.A. Linsenmeyer DRB, G.H. Creasey, B.G. Green, S.L.Groah, A. Joseph, L.K. Lloyd, I. Perkash, J.S. Wheeler. Bladder Management for Adults with Spinal Cord Injury: A Clinical Practice Guideline for Health-Care Providers. Spinal Cord Medicine. 2006:61.

44. International Perspectives on Spinal Cord Injury. WHO/ISCOS. 2013:250.

45. Takahashi S, Takei M, Nishizawa O, Yamaguchi O, Kato K, Gotoh M, et al. Clinical Guideline for Female Lower Urinary Tract Symptoms. Low Urin Tract Symptoms. 2016;8(1):5-29.
